# Efficacy of D-pigment dermocosmetic lightening product for solar lentigo lesions of the hand: A randomized controlled trial

**DOI:** 10.1371/journal.pone.0214714

**Published:** 2019-05-01

**Authors:** Federica Arginelli, Maurizio Greco, Silvana Ciardo, Gwendal Josse, Ana Beatris Rossi, Jimmy Le Digabel, Emmanuel Questel, Johanna Chester, Giovanni Pellacani

**Affiliations:** 1 Department of Dermatology, University of Modena and Reggio Emilia, Modena, Italy; 2 Pierre Fabre Dermo-Cosmétique, Clinical Skin Research Center, Toulouse, France; 3 Dermatology Department, Larrey University Hospital, Toulouse, France; Kinki Daigaku, JAPAN

## Abstract

Solar lentigo, benign lesions which mostly appear on chronically, sun-exposed surfaces, are associated with ageing. Patients are increasingly requesting a more uniform skin texture, especially for hands. Treatment options include dermoabrasion, intense pulsed light, cryotherapy, peelings, and laser therapy. Topical compounds can be employed, in alternative or associated with dermatologic procedures. The current study was designed to evaluate solar lentigo hyperpigmentation, skin architecture and clinician and patient assessments comparing a dermocosmetic lightening product (active) with a moisturizing product (control) according to clinical, digital and subjective analyses in 72 lesions over 12-month follow up period. Statistically significant differences were observed between the lesions treated with the active compared to the control in terms of papillary brightness (p = 0.03) and contrast (p = 0.03), and in the limitation of dermal-epidermal junction destructuring (p = 0.03) according to dermal-epidermal junction destructuring score at Reflectance Confocal Microscopy. Luminance (p = 0.04) and redness (p = 0.03) were improved at color analysis, and physician and patient evaluations favored the active in efficacy and patient satisfaction investigations. The dermocosmetic lightening product utilized in the current study proved to be more effective, according to clinical, digital and subjective analyses in reducing lesion hyperpigmentation, stabilizing the lesion skin architecture and increasing patient satisfaction compared to the control in a cohort of 36 subjects, over a 12-month period. Beside demonstrating the efficacy of this topical lightening product, we propose a “destructuring score”, which improves the robustness of solar lentigo’s evaluation, and can be used in future studies to standardize the quantitative comparisons of different treatment options.

## Introduction

Solar lentigo lesions are a widespread aesthetic and social concern, as they are associated with ageing. Solar lentigo lesions are benign and mostly appear on chronically, sun-exposed surfaces (face and scalp, dorsum of the hands, neckline and upper back), and are frequently present among the elderly population. Estimates suggest that >90% of Caucasians over 50 years old are affected [1].

Solar lentigo lesions are small, light to dark brown, flat and are usually smooth. At dermoscopy they appear as a faint to dark brown reticular pattern (fingerprinting) or a homogeneous pattern of pigmentation with sharply demarcated moth-eaten borders [2,3]. The most striking features of solar lentigo lesions using Reflectance Confocal Microscopy (RCM) are the polycyclic papillary contours at the dermal-epidermal junction (DEJ) and cord-like structures corresponding to the elongated rete ridges seen in histopathological analysis [4,5].

Patients are increasingly requesting a more uniform skin texture, especially for the hands. Treatment options include dermoabrasion, intense pulsed light (IPL), cryotherapy, peelings, and the current gold standard laser therapy. Topical compounds can be employed, as an alternative or associated with dermatologic procedures; the main active ingredients for skin lightening are hydroquinone, retinoids, azelaic and salycilic acids [6].

Solar lentigines have previously been assessed by RCM [7,8,9], but to date, investigations of topical dermocosmetic lightening products in solar lentigines have mostly been assessed with clinical scoring [10].

The current study was designed to evaluate solar lentigines hyperpigmentation, skin architecture and clinician and patient assessments comparing a dermocosmetic lightening product with a moisturizing product according to clinical, digital and subjective analyses in a large population with a long (12-month) application and follow up period.

## Materials and methods

### Study design

This was an intra-individual, randomized, hand controlled open label study conducted over a 12-month period in Modena, Italy with an allocation ratio of 1:1. Participants were enrolled into the study between 06 May 2013–26 June 2013. The study was conducted following the principles of the Declaration of Helsinki and approved by the Ethics Committee of the University of Modena and Reggio Emilia (prot. no. 1428, 20/13). All included participants gave written informed consent. The trial was registered (ClinicalTrials.gov NCT03457246), and the full trial protocol is available (S1 Protocol). As the local Ethics Committee did not require registration of the trial, registration was completed after patients’ enrolment, according to the editorial request. The authors confirm that there are no ongoing or related trials for this topical dermocosmetic lightening product.

**Fig 1:**
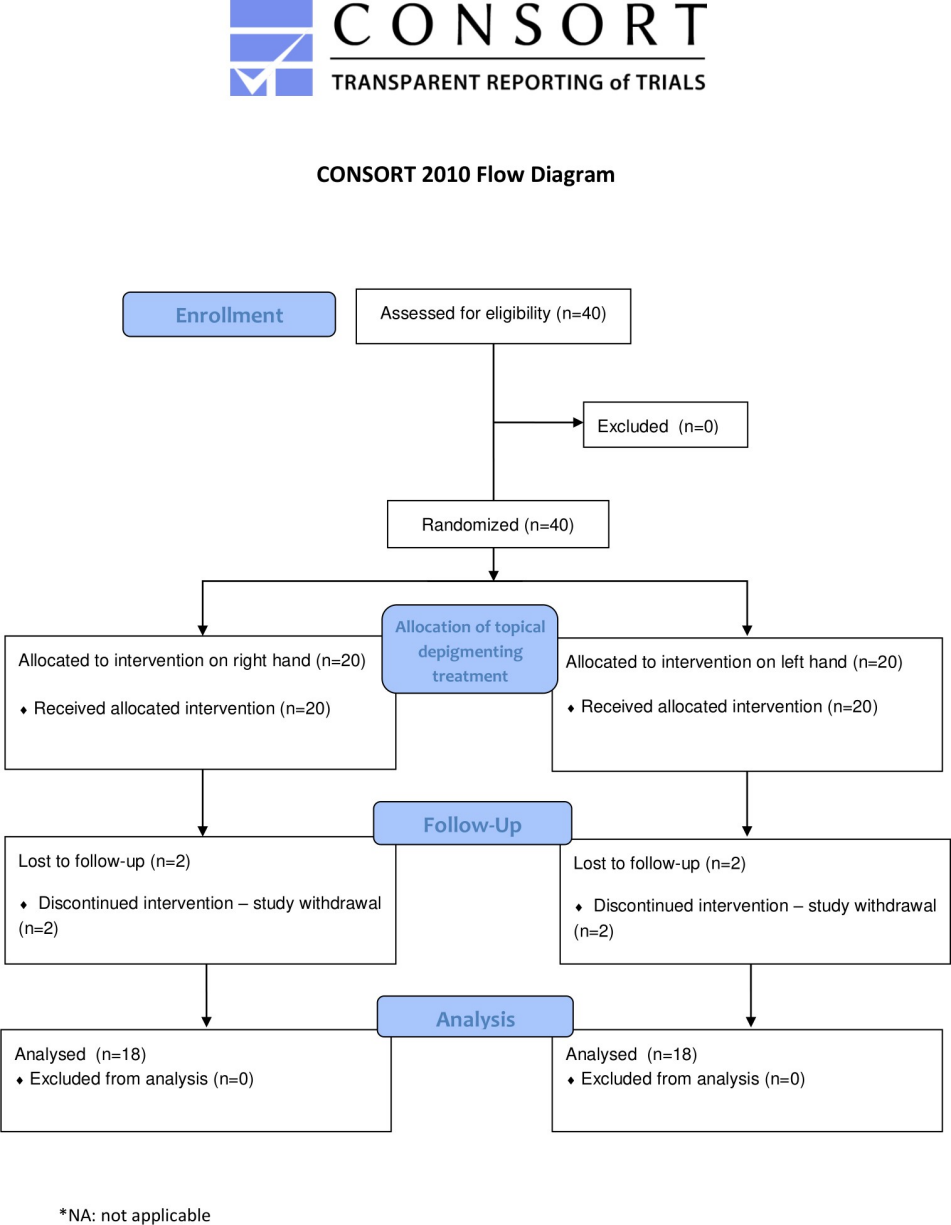
CONSORT flow chart of the study design.

Fig 1 shows the study design of the trial (Fig 1). Of the 40 patients assessed for eligibility, a target lesion per hand was chosen (one on the right hand, and one on the left hand). Treatment was then randomized according to which hand would receive topical treatment. A total of 36 subjects (72 lesions), were entered into final analysis, following the loss to follow-up of 4 patients (8 lesions). There were no changes to methods after trial commencement.

### Population

Patients were selected and enrolled by the study co-investigators following dermoscopic diagnosis of solar lentigo and according to an inclusion criteria specifying both male and female subjects 50 years and older, with skin phototype from II to IV according to Fitzpatrick’s classification and who gave written, informed consent to participate in the study. Subjects were excluded according to their intention to visit a region with a significant increase in sun exposure conditions compared to their usual place of residence (>7 days), women with childbearing potential, and subjects considered unlikely to remain compliant for the study period according to investigators’ judgement. The inclusion criteria related to solar lentigines were: the presence of at least 5 lentigines on the surface of each hand, characterized by the same severity; the target lentigo had to be moderately, markedly or extremely darker than the pigment of the peri-lesion area (4 mm), as assessed by investigators’ initial clinical evaluation. Subjects were also excluded according to any previous treatment for lentigines (especially laser treatment) within 8 weeks of enrolment, the presence of hyperpigmentation other than lentigines or other hypermelanosis (post-inflammatory hyperpigmentation, such as post-laser or chemical burning) and any other skin disorders or lesions on the dorsum of the hand. Any subjects with known hypersensitivity, allergy or intolerance to retinaldehyde or any other ingredient of the topical compounds were also excluded. Furthermore, diabetic subjects and subjects with chronic, progressive or systemic infectious diseases, which were considered as potential influencing factors for the study as judged by the investigator, were not included.

### Topical products

A randomization list was established prior to enrolment by the study sponsor and was concealed from the study investigators. At T0, the co-investigators allocated the study products to each subject according to the chronological order of arrival at the randomization visit, through a sequentially numbered letter allocation prepared by the study sponsor, without any restrictions. Following product assignment, investigators were no longer blinded to allocation. The tested active product was a topical dermocosmetic lightening product (D-Pigment, laboratoires Eau Thermale Avène, Pierre-Fabre, Boulogne, France), with a rich texture, containing phenylethyl resorcinol (0.5%, lightening agent), retinaldehyde (0.05%, a natural retinoid deriving from vitamin A) and tocopheryl glucoside (0.1%, photo-stable anti-oxidant precursor of vitamine E) as active ingredients. The reference product (RV2300G) was a moisturizer for cutaneous use (Hydrance, laboratoires Eau Thermale Avène, Pierre-Fabre, Boulogne, France), containing thermal water and shea butter.

The administration of the topical products was outlined by the protocol; application once a day (in the evening after hygiene), of the dermocosmetic lightening product on one hand and the moisturizer product on the other hand, for a period of 12 months. The products’ application was randomized to either hand. Patients were instructed to apply a dosage of each topical product equivalent to the size of a pea. Patients were given indications to avoid the application of sunscreens during the study period and any topical products on the day of the visits. The products were dispensed at each scheduled visit and investigators collected the products already consumed (empty tubes).

### Scheduled visits

**Fig 2:**
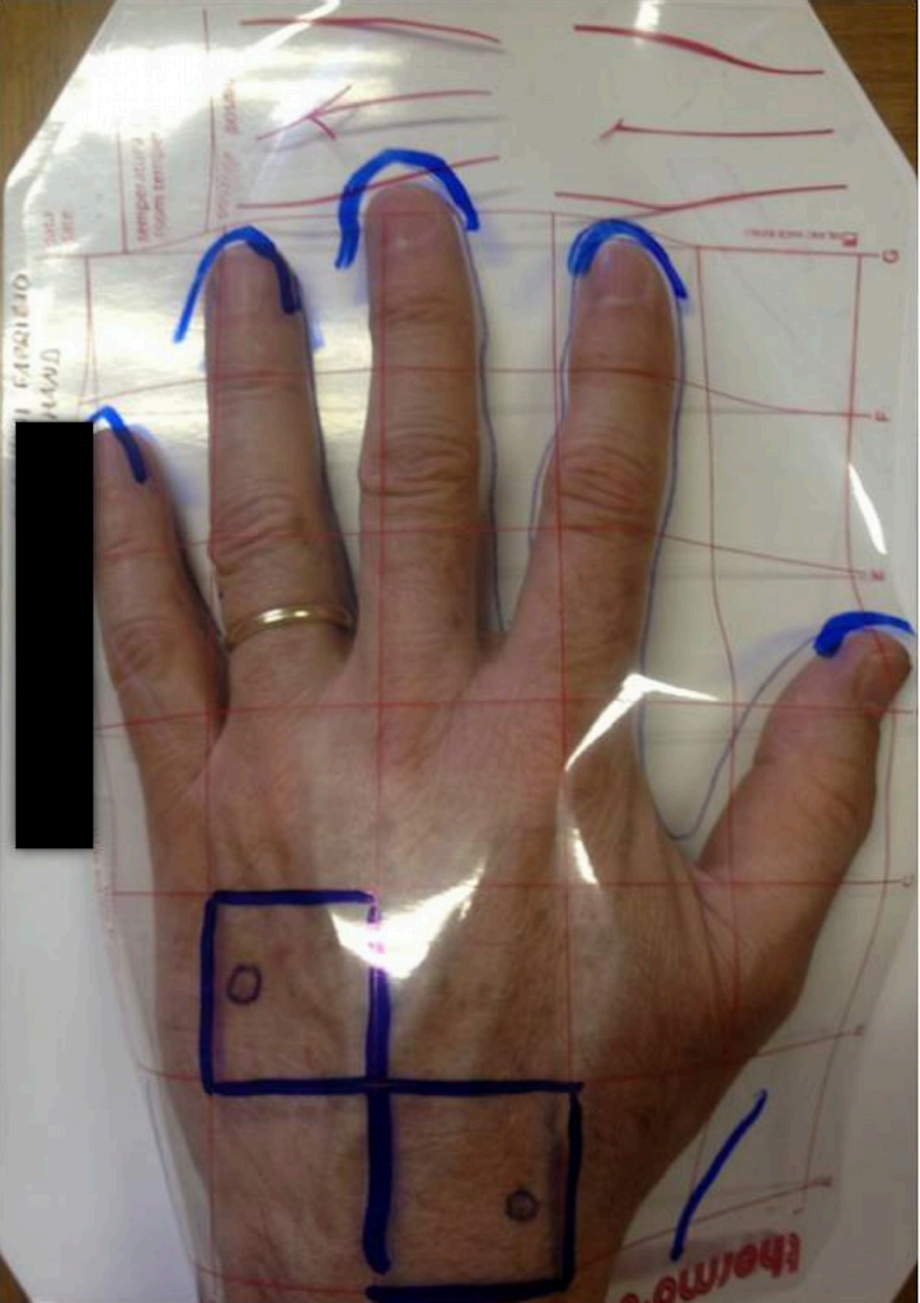
Digital standardized photo. Standardized photos were taken at T0 (baseline) with a plastic film placed over the hands to enable reproduction of the exact location of the target lesion during the follow-up.

Digital standardized photos, color calibrated imaging [11] and Reflectance Confocal Microscopy (RCM) acquisitions were performed during each visit; T0 (baseline), T3 (3 months), T6 (6 months), T9 (9 months) and T12 (12 months). Standardized photos were taken at baseline using a mask to standardize the same area of the hands and enable reproduction of the exact location of the target lesion during the follow-up (Fig 2).

### RCM evaluation

RCM images were acquired by Vivascope 3000 (MAVIG GmbH, Munich, Germany) at T0, T3, T6, T9 and T12. Vivascope 3000 generates horizontal sections of the skin corresponding to an area of 850 μm x 850 μm, using a near-infrared laser at 830 nm and operating at a power of less than 20 mW. For the study, a series of images were acquired from the center of each lesion, in sequence from the surface up to 150–200 μm in depth, each separated by 5 μm steps, generated by means of an automatic property software, Vivastack (MAVIG GmbH, Munich, Germany). The features evaluated included those observed at the dermal-epidermal junction (DEJ).

#### Intensity of papillary brightness

**Fig 3:**
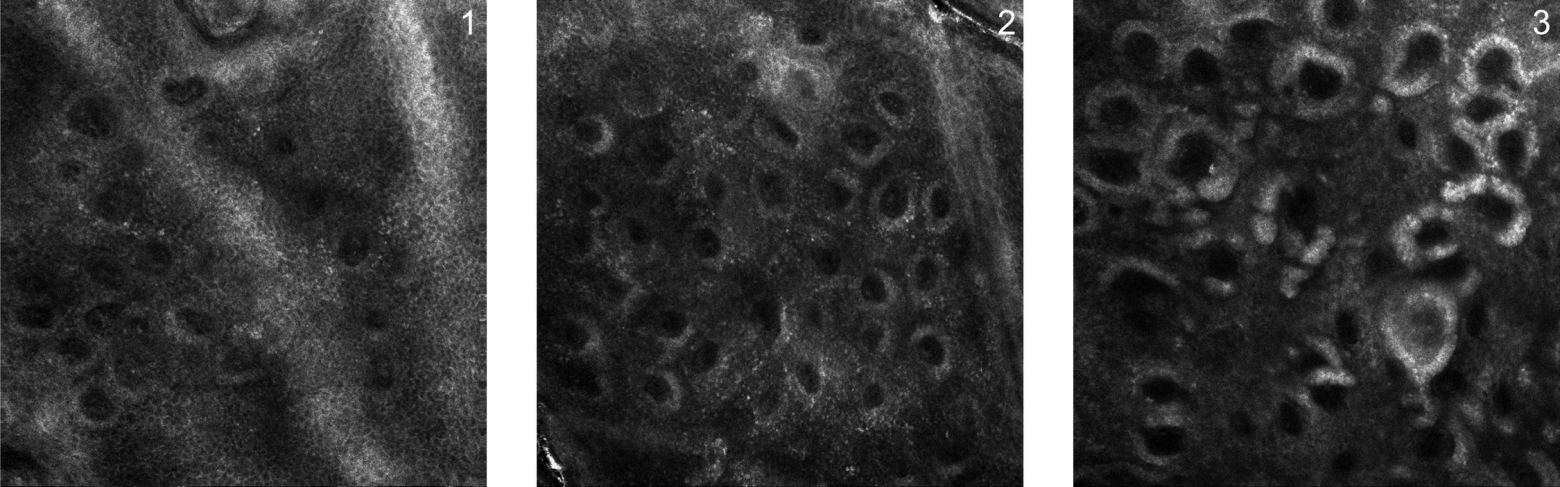
Intensity of papillary brightness. The intensity of papillary brightness is calculated through a numerical qualitative scale ranging from 1 to 3; 1: mild brightness resembling healthy skin; 2: intermediate brightness; 3: intense and sharp brightness of the papillary rim, due to a hyperpigmentation (melanin) of keratinocytes and melanocytes forming the rim.

A descriptive analysis was performed on the full sample, and frequencies and percentages of the intensity of papillary brightness were calculated at each time-point, according to a numerical qualitative scale ranging from 1 to 3; 1: mild brightness resembling healthy skin; 2: intermediate brightness; 3: intense and sharp brightness of the papillary rim, due to a hyperpigmentation (melanin) of keratinocytes and melanocytes forming the rim (Fig 3).

#### Papillary contrast

Images were analyzed with a software developed by Pierre Fabre’s imaging team to enable a semi-automated analysis of papillary contrast in the DEJ, at the level of 20 μm below the suprapapillary epidermal plateau. Papillary contrast was defined as the difference in brightness between the cellular ring around the papilla zone and the central dermal papilla zone, as described by Garrigo Lagarrigue et al. [12]

#### The dermal-epidermal junction (DEJ) destructuring score

The DEJ was assessed according to the most prevalent papillae features:

**(i) Round papillae:** Dark roundish and regular spaces surrounded by a rim of bright hyper-reflective cells (edged papillae), corresponding to pigmented keratinocytes and melanocytes.
**(ii) Scalloped rings:** Dermal papillae surrounded by a rim of small bright cells appearing as irregular bright rings sharply contrasting with the dark background.
**(iii) Enlarged interpapillary spaces:** Their presence or absence was evaluated at the DEJ, with respect to tightly packed papillae visible in solar lentigo and seborrheic keratoses.
**(iv) Polycyclic papillary contours:** Irregularly shaped dermal papillae, polymorphous and crowded, with annular and polycyclic bright contours surrounding dark irregular spaces. Their presence or absence was defined at the DEJ.

**Fig 4:**
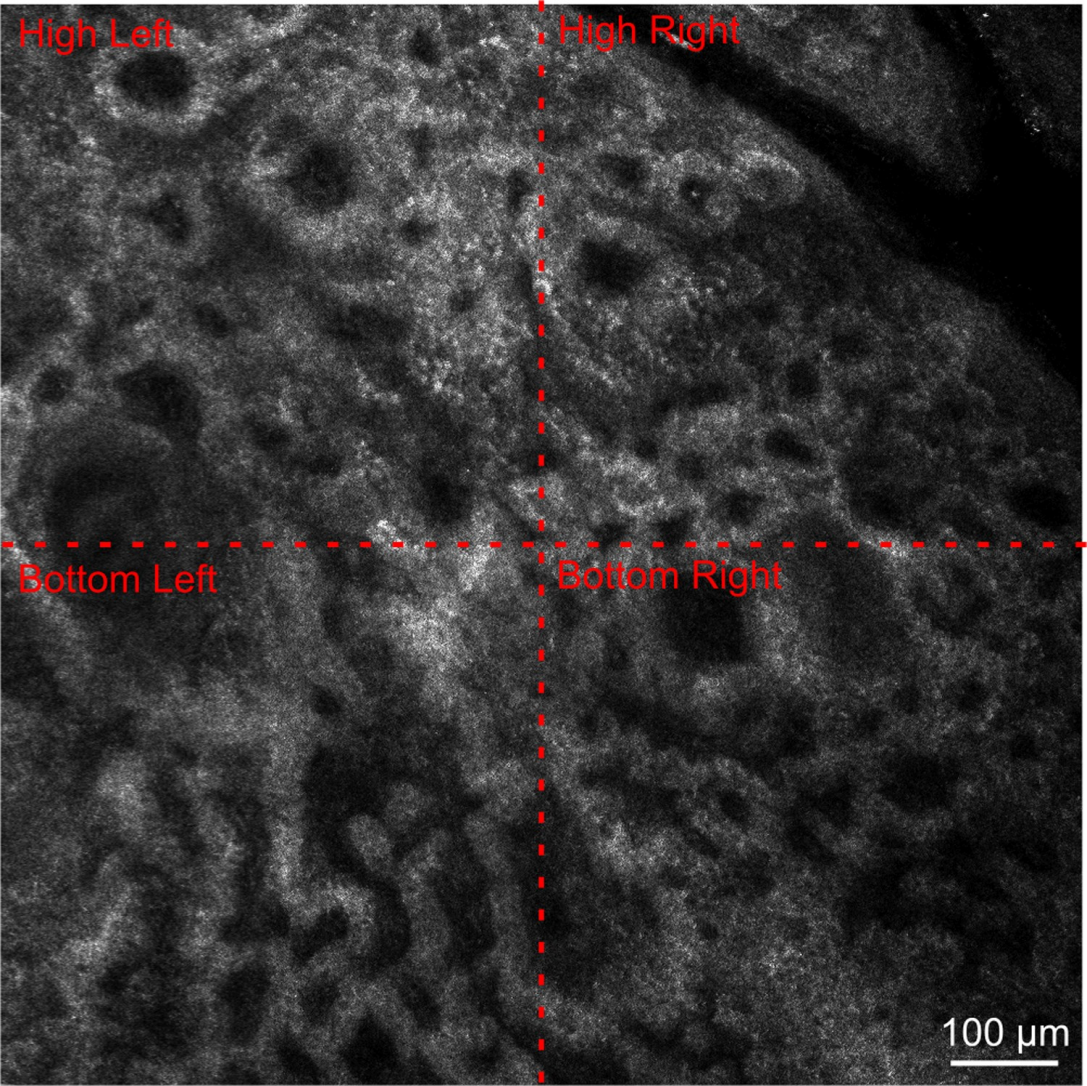
Papillae features assessed for the “DEJ destructuring” score. Evaluation of the most prevalent feature in each of the four quadrants of the image acquired (high left, high right, bottom left, bottom right).

In each of the four quadrants of the image acquired (upper right, upper left, lower right and lower left) (Fig 4), the most prevalent feature was noted. The authors propose a score for “DEJ destructuring” which includes the features of the lesion RCM image, weighted according to round papillae = 0; enlarged interpapillary spaces = 1; scalloped rings = 2; polycyclic papillary contours = 2. If these features are not present, their absence is considered 0. Each quadrant’s predominant feature is assigned a numeric value and the final score (the addition of each quadrant’s numerical value) is a value on the scale from 0–8.

### Color analysis

The target lesions were visualized by a high-magnification, skin imaging camera (BME; Artigues-pres-Bordeaux, France). The device, with a high-resolution (3 Mpixels), was color-calibrated as described by Vander Haeghen et al. [13] and optimized for color measurement on a 1 mm² area [11]. A manual delineation of the target lesion was performed on each image and the color parameters (CIE-Lab) were calculated. Image processing and color measurements were performed with a home-made software.

**Fig 5:**
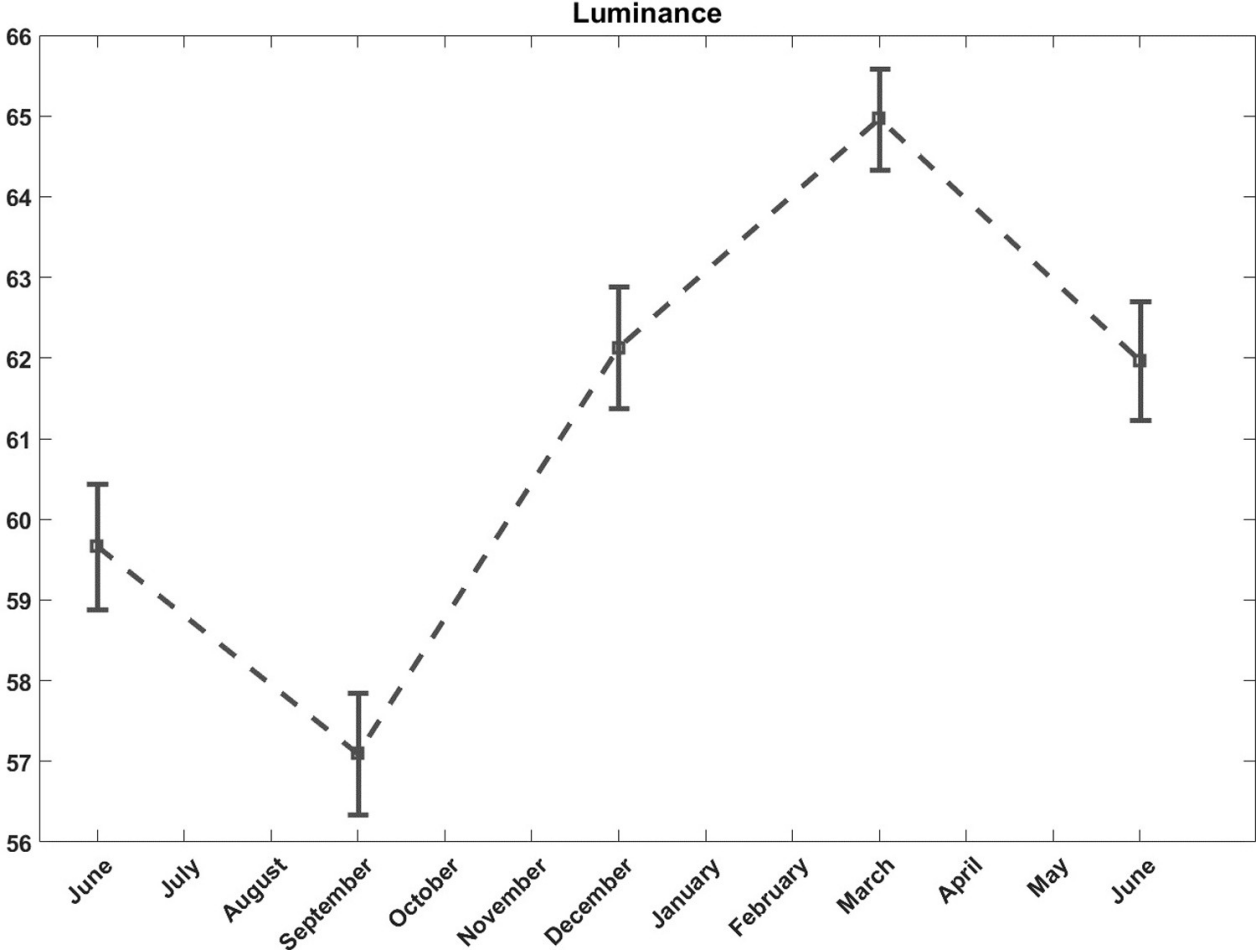
Color analysis. Seasonal effect of luminance of reference lentigines throughout the year.

As the skin color evolves seasonally, the results were corrected for seasonal variations, (Fig 5). The mean color value of the reference lentigines was used as a color reference at each time point to correct the seasonal effect.

### Physician analysis

Dynamic Physician Global Assessment (PGA) was performed to assess the direct effect of the topical treatment on the target lesion activity at T3, T6, T9 and T12. Scoring was recorded according to a comparison with baseline as: completely cleared, very significant clearance, significant clearance, moderate clearance, slight clearance and no change [14].

Tolerance for the use of the dermocosmetic lightening product was assessed by the investigator at T3, T6, T9, and T12 using a 4-points scale: 1: very good tolerance and no functional or physical signs at examination; 2: good tolerance with transitory functional signs and no physical signs at examination; 3: poor tolerance with persisting functional signs or physical signs from examination leading to modification of the conditions of administration but no application discontinuation; 4: very poor tolerance with functional and/or physical signs from examination leading to application discontinuation.

### Patient evaluation

A non-blinded patient efficacy assessment was performed for the global hand at T3 and T12, and noted in the patient file by the physician according to a 5-points scale: 0: completely improved, 1: mostly improved, 2: slightly improved, 3: not improved, 4: worsened.

Patient satisfaction was collected with a cosmetic agreement questionnaire, designed for the current study, with multiple-choice answers, and was administered at T3. Questionnaires were independently completed, without the intervention of the physician. The frequencies and the percentages of the evaluated parameters were calculated, comparing the dermocosmetic lightening product to the moisturizer product.

### Adverse events

Adverse events were defined as any noxious event, related or unrelated to the research or studied products. Events were graded as: mild, awareness of signs and symptoms, but easily tolerated; moderate, uncomfortable enough to cause interference with usual activity; and severe, incapacity with inability to work or do usual activity.

### Data collection and protocol deviations

All clinical data was collected and saved in a computerized database by the study sponsor, except for RCM data, which was collected and saved in a computerized database at the Department of Dermatology, University of Modena and Reggio Emilia, Modena, Italy. Due to technical issues with the quality of RCM images at T12, these evaluations were excluded from final analyses.

Major protocol deviations considered for the study included non-compliance with the inclusion or non-inclusion criteria, loss to follow-up, no assessment of the primary efficacy criterion and non compliance with products application (considered > 30% of missed application between 2 consecutive visits and > 30 days of sun exposure).

### Statistical analysis

Due to the lack of available data in literature, sample size calculation had no statistical rationale but a total of 30 subjects was considered satisfactory for study analyses. Assuming some subjects loss to follow-up over the entire study period of 12 months, a total of 40 subjects were recruited. Statistical analysis with the SAS software (Copyright 2014, SAS Institute Inc., Cary, NC, USA) was performed, including an analysis of covariance using a likelihood-based, mixed-effects model for repeated measures (MMRM) [15], from T0 to T12. Product, site and sequence were considered fixed factors (well-defined categorical variable), subjects were considered random factors (random sample of the entire population) and values at baseline were considered covariates (quantitative variable that can affect the outcome of the study).

## Results

Among the 40 recruited subjects, 4 were lost during the follow up period. Follow-up was finalized for all 36 included subjects on 09 June 2014. The data of the 36 subjects with a total of 72 lesions were considered according to originally assigned allocation. Among the 36 subjects, 1 subject missed the programmed visit at T6 and 1 missed the visit at T9. Of the 36 subjects, 34 were female (94.4%) and 2 were male (5.6%); the mean age was 61.4 ±8 years old (range from 50 to 79). None of the 34 females presented childbearing potential; 29 of them were in menopause and 5 had a previous history of ovariectomy and/or hysterectomy.

More than half (21–58.3%) of the enrolled subjects were phototype III (sometimes burns slightly, tans uniformly), 13 (36.1%) phototype II (usually burns slightly, tans slightly) and 2 (5.6%) phototype IV (burns slightly, always tans), (Table 1).

**Table 1:** Demographic data. Demographic data of the 36 patients enrolled into the study with complete follow-up.

Study population, *n*	36
Gender, *female (%)*	34 (94)
Mean age, *yrs (range)*	61.4 (50–79)
Phototype (Fitzpatrick classification), *n (%)*:	
II	13 (36)
III	21 (58)
IV	2 (6)

### RCM evaluation

**Fig 6:**
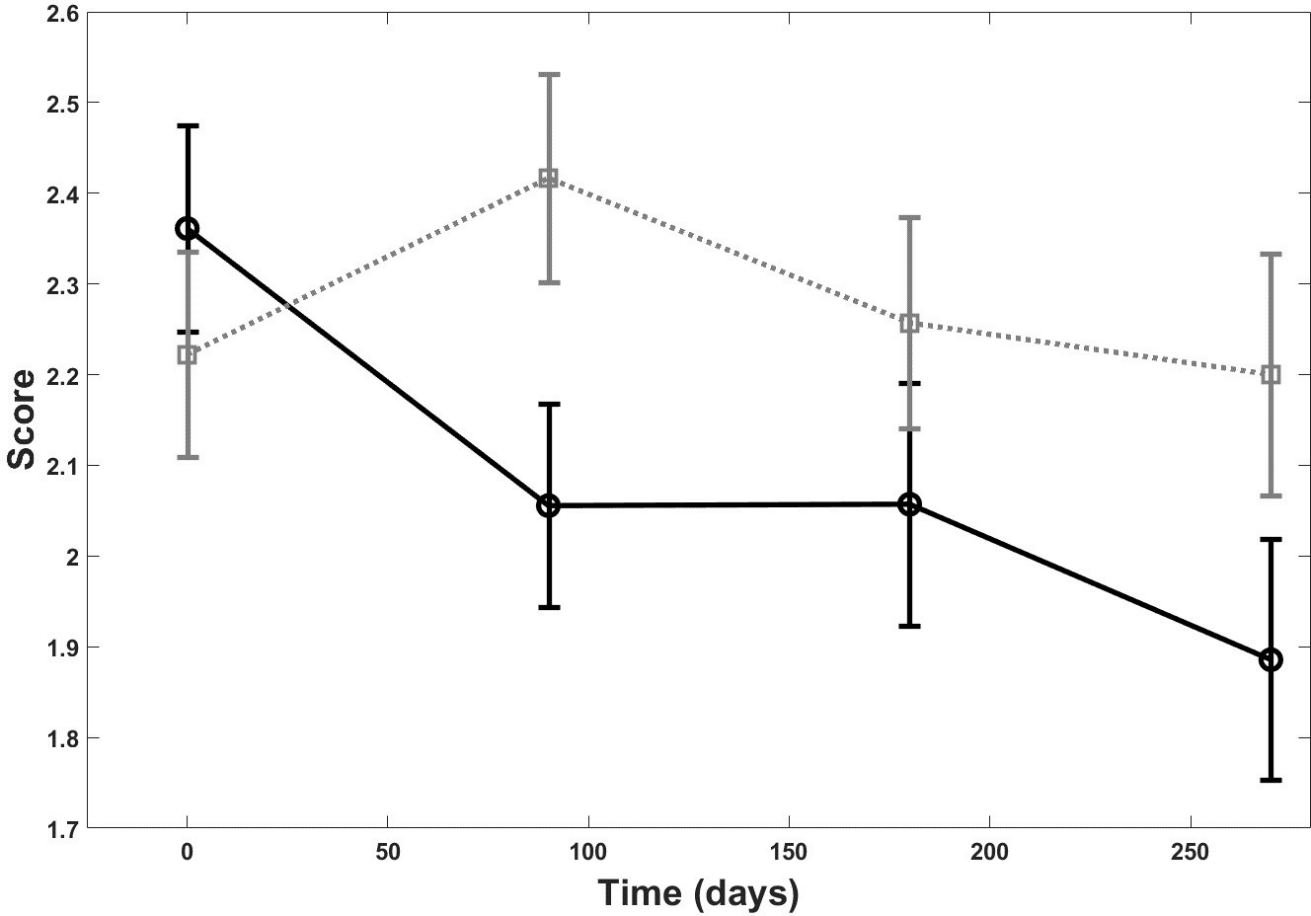
RCM and papillary brightness intensity. Papillary brightness intensity score at Reflectance Confocal Microscopy (RCM) over the 9 month follow-up period: dermocosmetic lightening product (solid line) and moisturizing product (dotted line).

**Fig 7:**
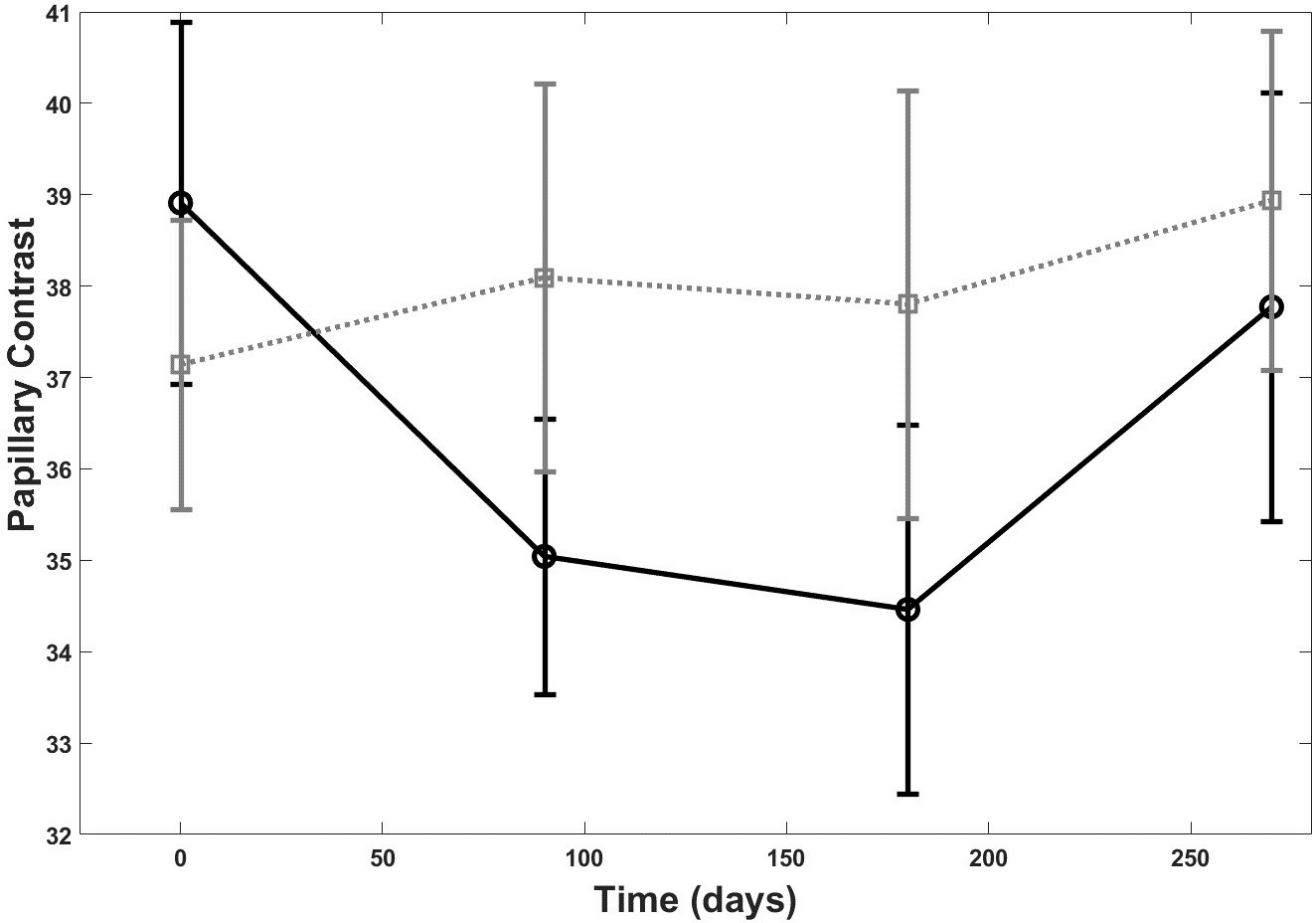
RCM and papillary contrast. Papillary contrast at Reflectance Confocal Microscopy (RCM) over the 9 month follow up period: dermocosmetic lightening product (solid line) and moisturizing product (dotted line).

**Fig 8:**
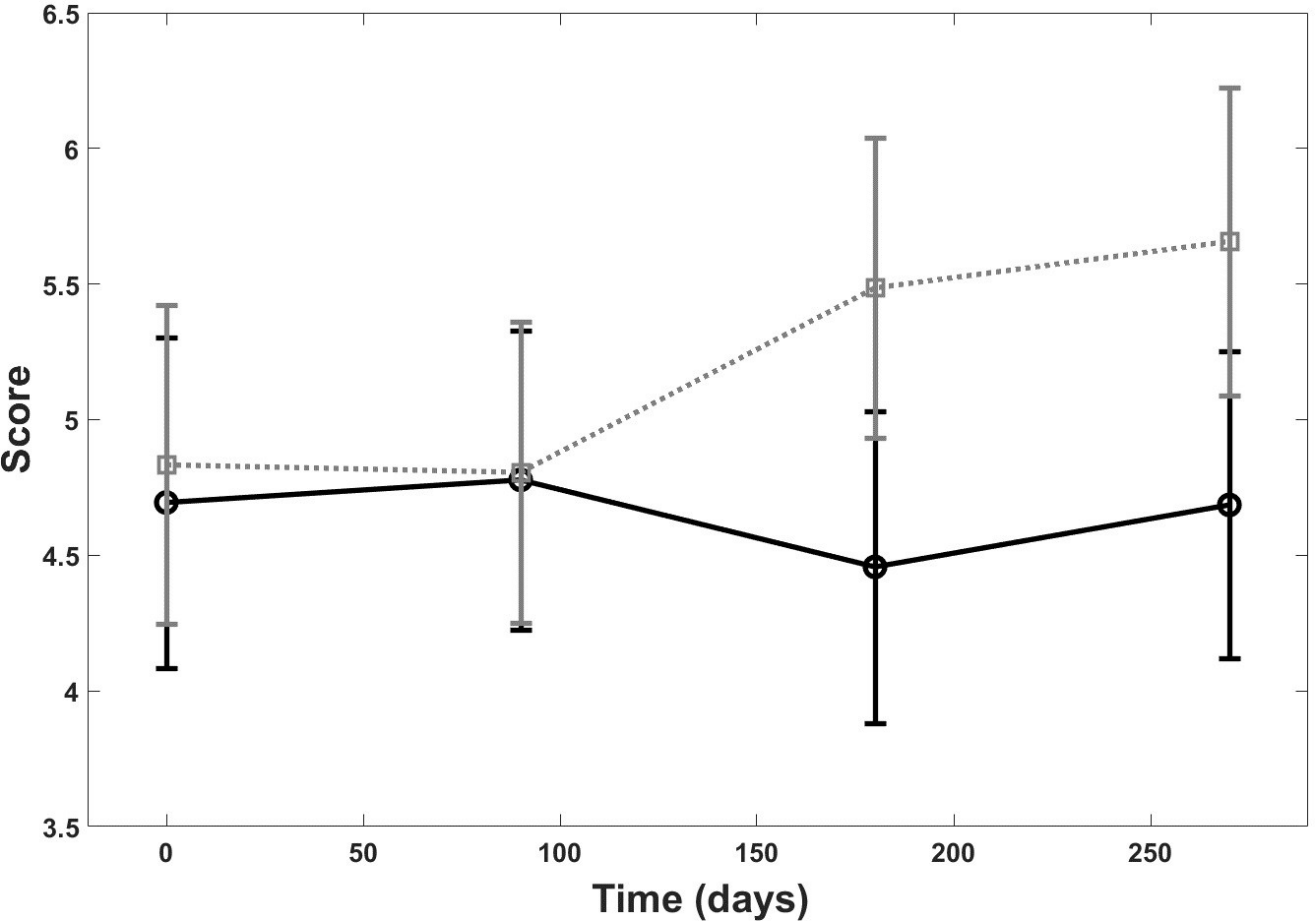
RCM and DEJ. DEJ destructuring score over the 9 month follow up period: dermocosmetic lightening product (solid line) and moisturizing product (dotted line).

A statistically significant difference was observed between the lesions treated with the dermocosmetic lightening product compared to the moisturizing product in terms of papillary brightness intensity at 3 months (p = 0.03). This improvement was maintained but was not statistically different from the moisturizing product during the follow-up period T6 and T9 (Fig 6). There was a statistically significant difference also between the groups for papillary contrast (p = 0.03), (Fig 7). The global effect of the dermocosmetic lightening product was found to be statistically significant in limiting the DEJ destructuring (p = 0.03), (Fig 8).

### Color analysis

**Fig 9:**
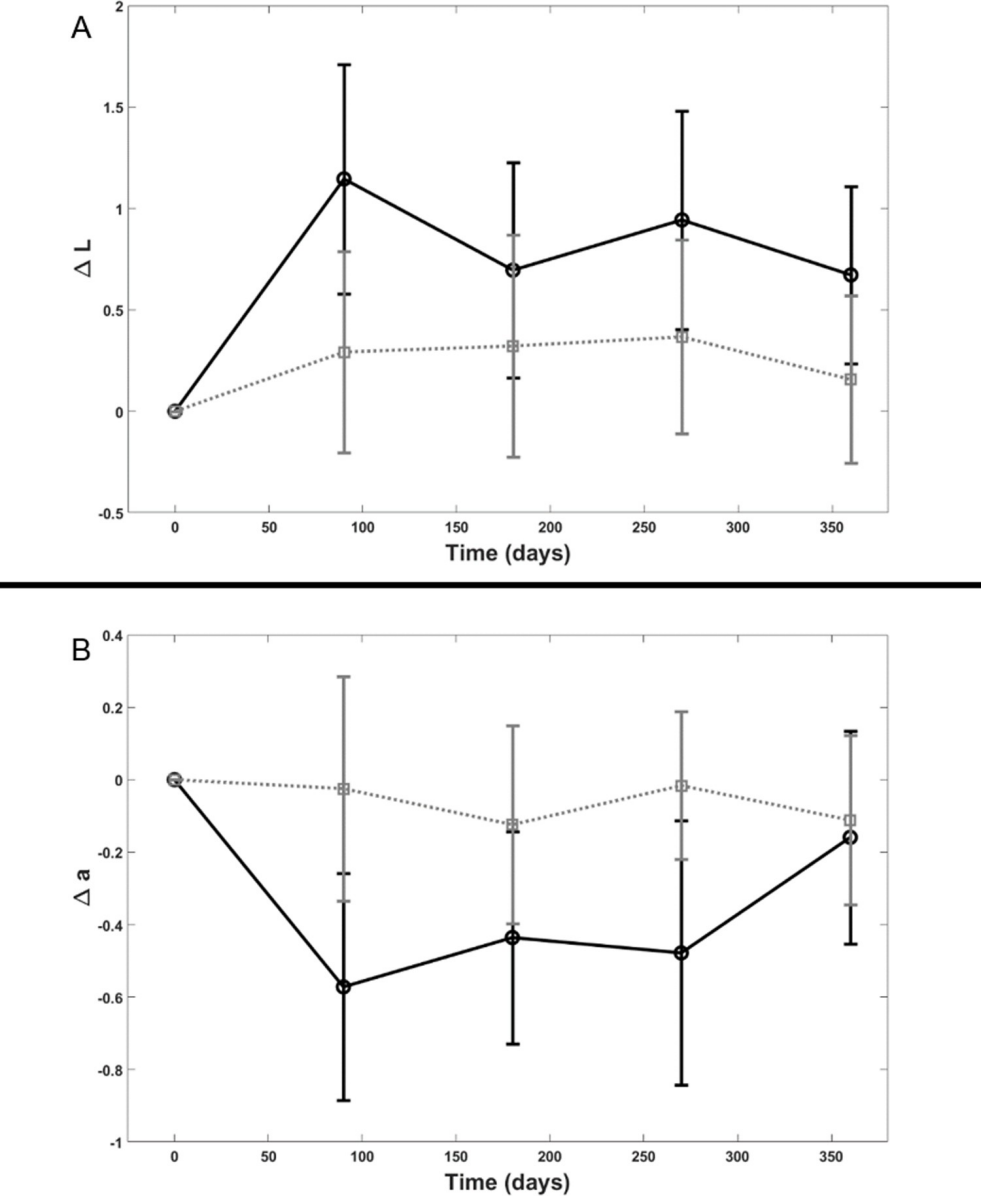
Color analysis. Color analysis for the lentigines luminance (Fig 9A) and the degree of redness (Fig 9B) for subjects treated with the dermocosmetic lightening product compared to moisturizing product; dermocosmetic lightening product (solid line) and moisturizing product (dotted line).

Color analysis revealed a statistically significant improvement in the lentigines luminance (p = 0.04) and the degree of redness (p = 0.03) for subjects treated with the dermocosmetic lightening product compared to moisturizing product (Fig 9).

### Physician analysis

**Fig 10:**
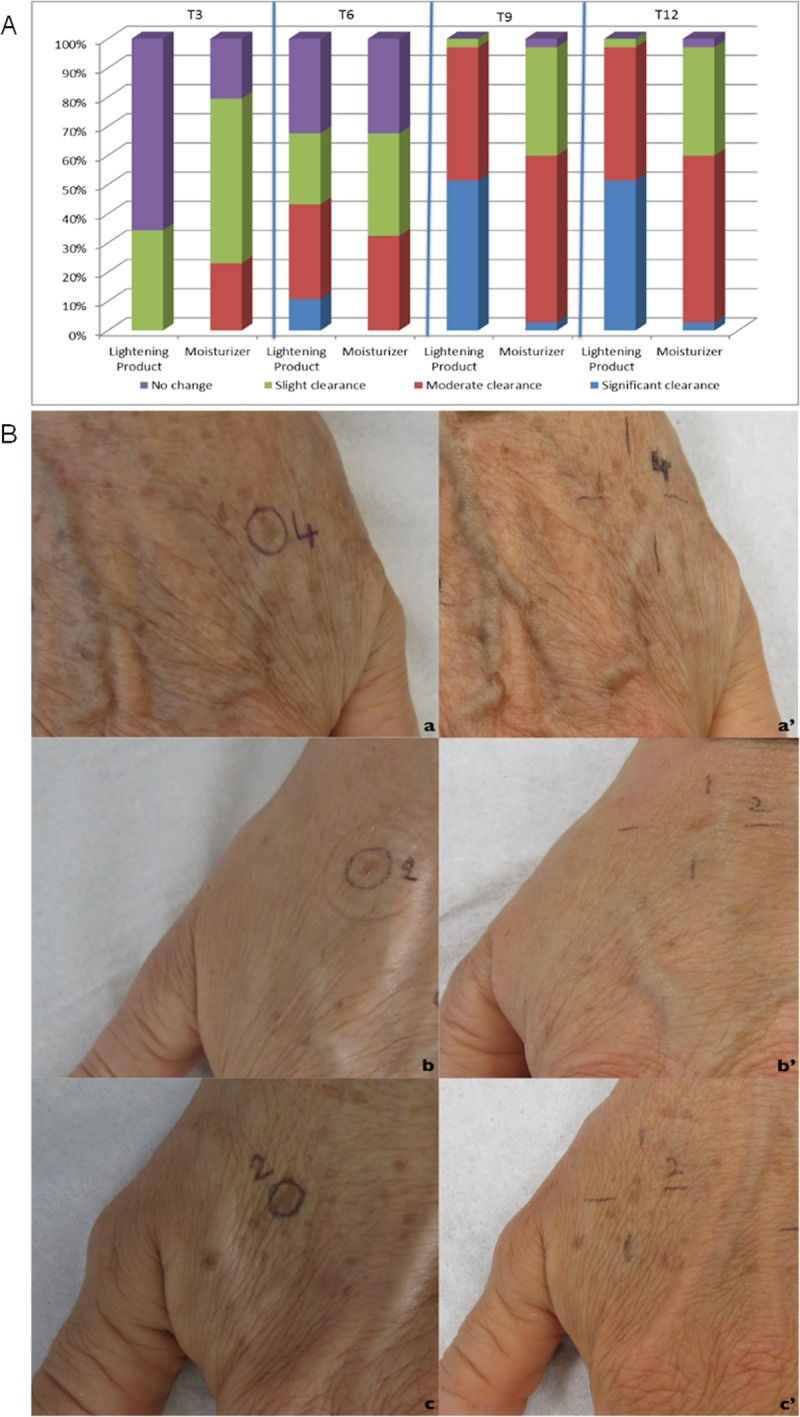
Physician global assessment. Fig 10A: clinical evaluation according to the dynamic Physician Global Assessment (PGA) of the 72 target lesions, presented according to the topical product applied. Fig 10B: clinical images of the 3 categories of improvement observed according to the PGA scale; slight clearance (a: T0, a’: T12), moderate clearance (b: T0, b’: T12) and significant clearance (c: T0, c’:T12), respectively.

The dermocosmetic lightening product was considered by the physician according to the PGA assessment to have a direct effect on skin improvement. The improvement was statistically greater than that measured for the moisturizing product at T6, T9 and T12, compared to T0 (p = 0.0036, p<0.001, p = 0.017, respectively), (Fig 10A and 10B).

Tolerance was evaluated for both products as “very good” for all subjects, at each scheduled visit after the baseline.

### Patient evaluation

**Fig 11:**
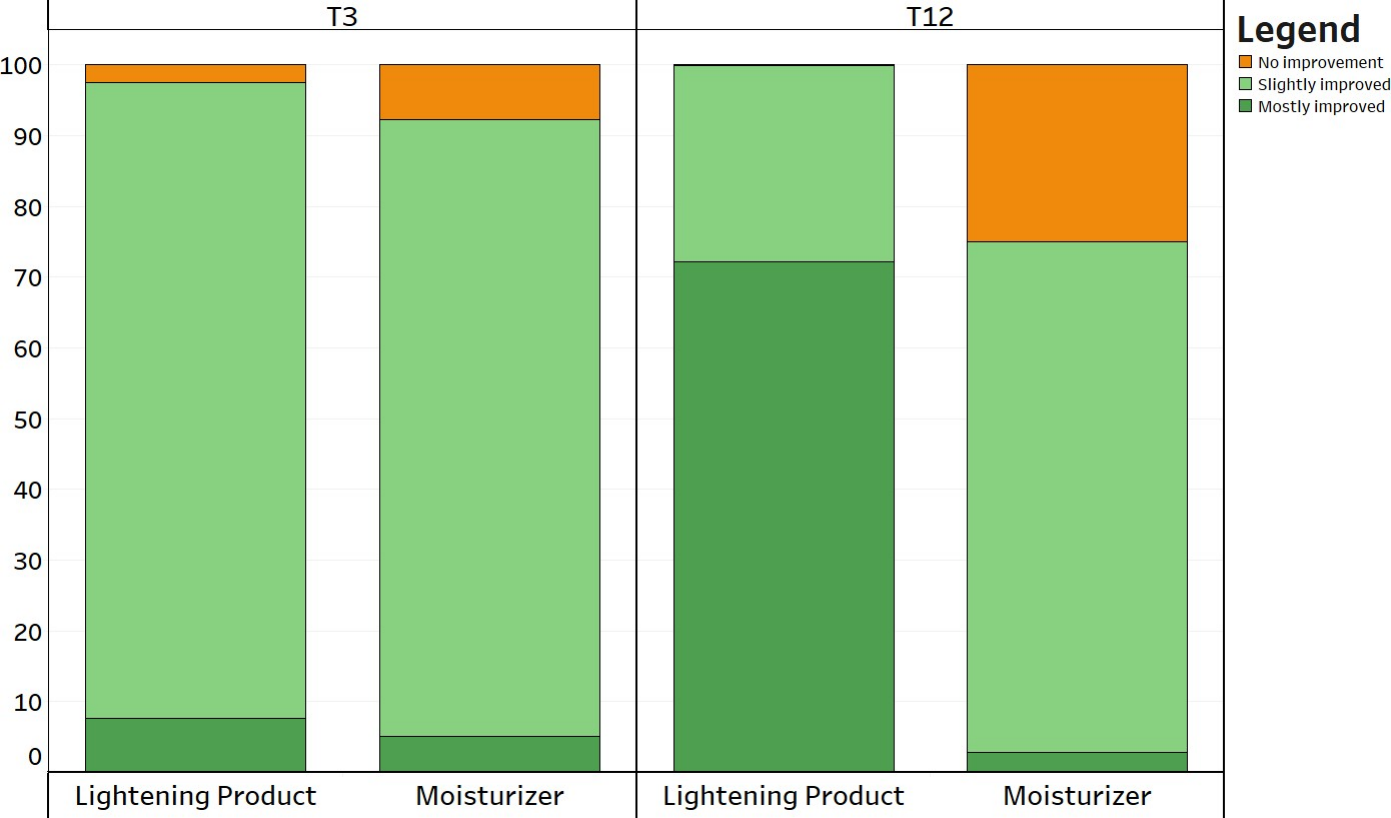
Patient evaluation. Patient efficacy assessment as either slightly improved, mostly improved or not improved.

Patient efficacy assessment revealed an improved overall efficacy for the dermocosmetic lightening product compared to the moisturizing product at long term (T12, p<0.001) but there was no difference observed in the short term (T3, p = 0.175), (Fig 11).

A statistically significant difference between the groups in terms of reported improvement in patient satisfaction on the target lesion for the depigmentation agent was found. A total of 38 of the 39 patients who responded to the questionnaire, reported being pleased or very pleased with the product after 3 months of application. Additionally, 87% of patients assessed the skin as more uniform and 92% agreed that the complexion and pigmentation was reduced. Other questionnaire results are outlined in Table 2.

**Table 2:** Patient satisfaction. Patient satisfaction outcomes for all patients after 3 months of the application of the dermocosmetic lightening product.

Questionnaire	Patients’ Responses, n (%)			
Overall satisfaction:	Not pleased	0 (0)		
	Fairly pleased	1 (2.6)		
	Pleased	22 (56.4)		
	Very pleased	16 (41)		
Product texture:	Unsatisfied	0 (0)		
	Satisfying	16 (41)		
	Very satisfying	23 (59)		
Skin penetration:	Unsatisfied	0 (0)		
	Satisfying	15 (38.5)		
	Very satisfying	24 (61.5)		
Sensation of the skin after product application:		Comfortable:	Fresh & clean:	Sticky:
	No	0 (0)	4 (10.3)	34 (87.2)
	Yes, a little	3 (7.7)	9 (23.1)	5 (12.8)
	Yes	24 (61.5)	23 (59)	0 (0)
	Yes, absolutely	12 (30.8)	3 (7.7)	0 (0)
Skin following product application:		Skin more uniform:	Complexion and pigmentation reduced:	Intensity of the spots attenuated:
	No	5 (12.8)	3 (7.7)	1 (2.6)
	Yes, a little	13 (33.3)	21 (53.8)	22 (56.4)
	Yes	16 (41)	11 (28.2)	13 (33.3)
	Yes, absolutely	5 (12.8)	4 (10.3)	3 (7.7)
Comparison to other products previously used:	Inferior	0 (0)		
	Equivalent	14 (53.8)		
	Better	8 (30.8)		
	Not applicable*	4 (15.4)		

* not applicable: patients without previous product experience.

### Adverse events

There were few adverse events during the study. None were related to the study products and most were of mild intensity. There were no serious or severe adverse events during the study.

### Protocol deviations

There were 4 patients lost to follow up (preprogrammed visits not performed with subsequent missing evaluation of primary criterion). Furthermore, 4 major protocol deviations were registered: 2 cases of excessive sun exposure (>30 days throughout the year) and 2 missed doses application (>30%) at final follow-up.

## Discussion

Clinical studies designed to evaluate the lightening effect of active ingredients on solar lentigines usually use clinical scoring as the single endpoint [10,16–18]. In order to respond to the need for a more robust approach in measuring the effectiveness of a lightening topical product, the present study proposes a unique design including clinical, digital and subjective analyses. Further, most studies on lentigines are performed over a short period of up to 3 months [17,19–22], and with a limited number of enrolled subjects, with an average of 10–20 subjects per study [19,23]. The current study design included a longer follow up of 12 months, enabling the consideration of seasonal impact on lentigines pigmentation on a relatively large population cohort of 36 subjects and a total of 72 lentigines.

Various options are available for the removal of solar lentigines, such as lasers (CO_2_, Q-Switched Nd-YAG, Ruby), intense pulsed light, cryotherapy and topical bleaching actives [6,23–34]. The advantages of a topical product for solar lentigines’s treatment are numerous. Dermocosmetics have no associated risks, are non-invasive and do not require a healing period after treatment, therefore reducing the risk of post-inflammatory hyperpigmentation, as compared to cryotherapy, IPL and laser treatments. Another important advantage, deriving from the application of a cream, is the possibility to treat the whole hand (lentigines and peri-lesion areas) for a more uniform appearance, instead of the ablation of the single lesion obtained with other treatment options. This can provide a “preventive” action, as it has been described that there is subclinical alterations observed in the lentigines peri-lesional area [35]. Further, the depigmenting agents employed in this study, including phenylethyl resorcinol 0.5%, retinaldehyde 0.05% and tocopheryl glucoside 0.1%, have been proven to be well tolerated into the long term [36–38]. Regular application is therefore safe for the treatment and maintenance of a chronic aesthetic issue, such as solar lentigines. Additionally, the application of a topical treatment is a common daily gesture, cheaper than other interventions, and is a useful preparative/maintenance therapy associated with more invasive procedures.

Color measurement can quantify the visual perception of colors. It has been shown to be a precise and robust technique for skin lightening efficacy evaluation [8,22,27,39]. Pierre Fabre imaging team previously showed that precise skin color measurements can be performed using a high-magnification color-calibrated camera, also used to follow up lentigines [20]. The present study includes this new technology. The luminance and redness were both found to be improved in association with the dermocosmetic lightening product throughout the follow-up. Additionally, these chromametric findings also corresponded well with patients’ satisfaction. Along with the general improvement of luminance and redness associated with the dermocosmetic lightening product, patients reported as early at 3 months, an attenuation of the spots’ intensity.

The visualization of the skin structures and evaluation of pigment modulation throughout the study follow-up was performed with RCM, an established non-invasive imaging tool enabling the visualization of skin structures at various depths, with an almost histologic resolution [40,41]. Its application in studies dedicated to aesthetic improvements is valuable due to the avoidance of skin biopsy [42]. RCM is increasingly employed in the diagnostic field and has become an important device to assess the follow-up of treatments for benign lesions [7]. In the case of solar lentigines, it has been proven to be effective in in situ pigmentation evaluation [8]. To our knowledge this is the first study that evaluates the lightening efficacy of a topical treatment through a standardized analysis of RCM images in lentigines.

Changes in papillary brightness and papillary contrast, both assessed with RCM, were also reflected in the clinical assessment of visible changes in lentigo’s pigmentation. According to RCM analysis, the employment of the dermocosmetic lightening product highlighted a statistically significant difference of papillary brightness intensity at T3 compared with the moisturizing product and accentuates that the main action of improvement is verified within a 3 month period, with a further limited improvement observed after 6 months. The seasonal effect over the 9 month period is clearly visible by the moisturizing product (dotted line, Fig 6). From a clinical point of view, the non-blinded to treatment assignment physician assessment, reported an improvement in the target lesion treated with the dermocosmetic lightening product, both at T6 and T9, in comparison to baseline. These results were also well translated into patient’s satisfaction assessment throughout the study. At the first patient survey (T3), the intensity of the target lesions were evaluated as attenuated by 97% of the subjects. This was preserved at the final questionnaire (T12) as the overall efficacy of the dermocosmetic lightening product for the whole hand was assessed as significantly improved, compared to the moisturizing product.

In the current study, the authors propose a quantitative ‘destructuring score’ in order to measure the disorganization of the DEJ associated with solar lentigines. The disorganization of the DEJ of lentigo lesions has previously been described with RCM [4,8,9]. Pollefliet et al. showed the progression of solar lentigines over time with RCM. The introduction of a score can provide a more robust comparison between treatment options in future studies. The current study have shown the quantitative limitation of natural DEJ destructuring of solar lentigines, associated with sun exposure and ageing, with the use of the dermocosmetic lightening product compared to the moisturizing product. This was demonstrated by a stabilization of the destructuring score associated with the dermocosmetic lightening product, compared to the deterioration of the DEJ, with indications of more disorganization, observed in the hand treated with the moisturizing product. No adverse events occurred during the study.

However, the study has some limitations. There was a potential ascertainment bias as investigators, data manager and subjects were not blinded to topical product allocation throughout the follow-up and data analyses periods. Further, due to the sample size, data should be interpreted with caution given the imprecision inherent in small sample studies. The inclusion of more than one lesion could have assisted in obtaining more robust data. Additionally, the study protocol limited patients’ sun exposure and application of sunscreen. Finally, adverse events were not collected according to a standardized method but were simply reported in the case report form according to common events associated with topical products reported in literature.

The authors hypothesize that with the integration of a sunscreen daily application, results could be expected to improve, especially in association with DEJ destructuring. Additionally, future studies could evaluate the benefit of the associated use of dermocosmetics with other therapeutic approaches and a comparison of other treatment options for lentigo into the long term. These would be useful for an accurate comparison of efficacy, patient satisfaction and economic impact to better define treatment cost-effectiveness.

## Conclusions

The dermocosmetic lightening product tested in the study proved to be more effective, according to clinical, digital and subjective analyses in reducing lesion hyperpigmentation, stabilizing the lesion skin architecture and increasing patient satisfaction compared to a moisturizing product in a large patient cohort over a 12 month period. The introduction of a “destructuring score”, as proposed by the current authors, could improve the robustness of future evaluations of solar lentigines treatment options, by providing objective quantitative measurement that correlates with clinical assessments.

## Supporting information

S1 Protocol Full trial protocol, study code: RV4280A2012607. (PDF)

S1 Checklist Consort checklist. (PDF)
