# Supplementary material for: Efficacy of D-pigment dermocosmetic lightening product for solar lentigo lesions of the hand: A randomized controlled trial
Source: PLoS One. 2019 May 1;14(5):e0214714. doi: 10.1371/journal.pone.0214714 (PMC6493707; doi:10.1371/journal.pone.0214714)
Supplement: S1 Protocol — (PDF) [file pone.0214714.s001.pdf]

Sponsor : PIERRE FABRE DERMO-COSMÉTIQUE
45 place Abel Gance
92654 BOULOGNE
FRANCE

Study Code: RV4280A2012607

Internal Study Code: HDpb_2012_AVEN_060_RV4280A2012607

Sponsor : PIERRE FABRE DERMO-COSMÉTIQUE
45 place Abel Gance
92654 BOULOGNE
FRANCE

CLINICAL STUDY PROTOCOL

Efficacy and tolerance of D-pigment versus moisturizer in hands' lentigo lesions with or without laser therapy

A randomized, intra-individual hand controlled open label study

Study Product	D-pigment – RV4280A
Pierre Fabre Study Code :	RV4280A2012607
Internal Study Code :	HDpb_2012_AVEN_060_RV4280A2012607
Sponsor's Representative :	Emmanuel QUESTEL<br>Hôtel Dieu<br>2, rue Viguerie<br>31 025 Toulouse Cedex 3 France<br>Phone : +33 (0)5 62 48 85 05<br>Fax : +33 (0)5 62 48 85 99<br>E-mail : emmanuel.questel@pierre-fabre.com<br><br>Catherine JEAN DECOSTER<br>Les Cauquillous<br>81 506 Lavaur Cedex<br>Phone : +33 (0)5 63 58 97 18<br>E-mail : catherine.jean.decoster@pierre-fabre.com
Principal Investigator :	Pr Giovanni PELLACANI<br>Department of Dermatology<br>University of Modena and Reggio Emilia<br>Via del pozzo 71<br>41100 MODENA<br>Phone : +39 (0)59 422 4264<br>Fax : +39 (0)59 4224271<br>E-mail : pellacani.giovanni@unimore.it

Version n° 1 - Date of version : 21/01/2013

CONFIDENTIAL DOCUMENT

The information contained in this document is confidential and is the property of the sponsor PIERRE FABRE DERMO-COSMÉTIQUE. This information is given for the needs of the trial and must not be disclosed without prior written consent of the sponsor PIERRE FABRE DERMO-COSMÉTIQUE.
Persons to whom this information is given for the needs of the study must be informed that it is confidential and must not be disclosed.

APPROVAL FORM

Study : RV4280A2012607

(Internal Study Code : HDpb_2012_AVEN_060_RV4280A2012607

PROTOCOL Version N°1 dated : 21/01/2013

Sponsor's representative(s) :

- Avène General Manager

Name : Alexandre BRENNAN Date : Signature :

- Monitor

Name : Emmanuel QUESTEL Date : Signature :

- Statistician

Name : Christophe LAUZE Date : Signature :

- Avène Medical Officer

Name : Catherine JEAN DECOSTER Date : Signature :

Study Principal Investigator :

Name : Pr Giovanni PELLACANI Date : Signature :

INVESTIGATOR SIGNATURE FORM

Study : RV4280A2012607

(Internal Study Code : HDpb_2012_AVEN_060_RV4280A2012607

PROTOCOL Version N°1 dated : 21/01/2013

By my signature below, I, Dr. ______________________, hereby confirm that I agree :

- to conduct the trial described in the protocol n° RV4280A2012607 dated 21/01/2013 in compliance with GCP, with applicable regulatory requirements and with the protocol agreed upon by the sponsor and given approval/favourable opinion by the Ethics Committee ;
- to document the delegation of significant study related duties and to notify the sponsor of changes in site personnel involved in the study ;
- to comply with procedures for data recording and reporting ;
- to permit monitoring, auditing and inspection ;
- to retain the trial-related essential documents until the sponsor informs these documents are no longer needed.

Furthermore, I hereby confirm that I will have and will use the availability of adequate resources, personnel and facilities for the conduct of this trial.

Date : Signature :

Table of contents :

SYNOPSIS **8**
STUDY FLOW CHART **15**
1. INTRODUCTION AND STUDY RATIONALE **17**
1.1. Target pathology and therapy **17**
1.2. Information on the test product **17**
1.3. Study rationale **18**
1.4. Overall risk and benefit assessment **18**
2. STUDY OBJECTIVES **18**
2.1. Primary Objective **18**
2.2. Secondary objectives **18**
3. ETHICAL CONSIDERATIONS RELATING TO THE STUDY **19**
4. STUDY DESIGN **20**
4.1. Overall description **20**
4.2. Discussion of the study design **20**
5. STUDY POPULATION **20**
5.1. Inclusion criteria **20**
5.2. Non inlusion criteria **21**
5.3. Specific requirements **22**
5.4. Number of subjects **22**
5.5. Recruitment modalities **22**
5.6. Subject identification **22**
5.7. Withdrawal criteria **22**
5.8. Replacement of subjets **22**
5.9. Post-study exclusion period **23**
6. STUDY PRODUCTS **23**
6.1. Source, presentation and composition of investigational products and associated product **23**
6.1.1. Test product 23
6.1.2. Reference Product 23
6.2. Packaging and labelling **23**
6.2.1. Packaging 23
6.2.2. Labeling 24
6.3. Distribution to centre and storage **24**
6.4. Allocation of products and dispensation to subjects **24**
6.5. Products administration **24**
6.5.1. Duration of products application 24
6.5.2. Route, dosage and conditions of administration 25
6.6. Accountability on site and return/destruction of investigational products and associated product **25**
6.7. Recall of investigational products **25**
7. CONCOMITANT TREATMENTS **25**
7.1. Prohibited treatments, products and particular practice concerning the skin **25**
7.2. Authorised treatments, products and particular practice concerning the skin **26**
8. EVALUATION CRITERIA **26**
8.1. Efficacy assessment **26**
8.1.1. Primary Criterion 26
8.1.2. Secondary criteria 26
8.1.2.1. Criteria assessed on global areas *26*
8.1.2.2. Criteria assessed on target lesions *27*
8.1.2.3. Criteria assessed by the subjects *29*
8.2. Safety assessment **29**
8.2.1. Adverse Events 29

8.2.2. Local tolerance 29
8.3. Compliance 30
9. STUDY PROCEDURES 30
9.1. Testing conditions 30
9.2. Visit 1 – Inclusion visit (Day 1) 30
9.3. Visit 2 – intermediate visit (Day 90 ± 8 days) 31
9.4. Visit 3 – intermediate visit (Day 180 ± 8 days) 31
9.5. Visit 4 – intermediate visit (Day 270 ± 15 days) 31
9.6. Visit 5 – End-of-study visit (Day 360 ± 15 days) 32
10. ADVERSE EVENTS : DEFINITION AND REPORTING 32
10.1. Adverse events 32
10.1.1. Definition 32
10.1.2. Grading of Adverse Events 32
10.1.3. Reporting of Adverse Events 32
10.2. Serious Adverse Events (SAE) 33
10.2.1. Définition 33
10.2.2. Reporting of SAE 33
10.2.3. Follow-up of SAE 33
10.2.4. Suspicions of Serious adverse effects occurring after the study 34
10.3. adverse events related to the study products requiring medical treatment or likely to be serious 34
10.4. New Events 34
10.5. Sponsor's responsibilities for safety reporting purposes 34
11. DATA COLLECTION AND STUDY MONITORING 34
11.1. Data Collection 34
11.1.1. Case Report Form 34
11.1.2. Source Documents 35
11.1.3. Computerized data 35
11.2. Study Monitoring 35
11.2.1. Monitoring visits 35
11.2.1.1. Site preselection visit 35
11.2.1.2. Initiation visit 36
11.2.1.3. Follow-up visits 36
11.2.1.4. Closing visit 36
11.2.2. Direct access to source documents 36
12. DATA MANAGEMENT 36
12.1. Data Entry 36
12.2. Data Review 37
12.3. Data storage 37
12.4. Database lock 37
13. STATISTICAL ANALYSIS 37
13.1. General considerations 37
13.2. Sample size 37
13.3. Protocol deviations 37
13.4. Data sets analysed 38
13.5. Handling of dropouts and missing data 38
13.6. Statistical Methods 38
13.6.1. Description at baseline 38
13.6.2. Efficacy analysis 39
13.6.2.1. Primary criterion 39
13.6.2.2. Secondary criteria 39
13.6.3. Safety Analysis 40
13.6.3.1. Adverse Events 40
13.6.3.2. Concomitant treatments 40
13.7. Compliance 40
14. GENERAL ETHICAL CONSIDERATIONS 40
14.1. Ethical conditions 40

14.2. Ethics committee and legal requirements **41**
14.3. Subject's information leaflet and informed consent form **41**
14.4. Personal data protection **41**
14.5. Insurance policy **41**
15. ADMINISTRATIVE PROCEDURES **42**
15.1. Protocol amendment **42**
15.2. Source documents, investigator's file storage **42**
15.3. End of the study **42**
15.3.1. Definition of the end of study 42
15.3.2. Early study termination 42
15.3.2.1. Early study termination decided by the sponsor *42*
15.3.2.2. Early study termination decided by the Competent Authorities *43*
15.4. Audit **43**
15.5. Inspection **43**
15.6. Confidentiality **43**
15.7. Clinical study report **43**
15.8. Study results communication **43**
15.9. Study results publication **43**
16. REFERENCES **45**
17. APPENDICES **46**
17.1. Declaration of Helsinki **47**
17.2. Serious Adverse Event (SAE) / Pregnancy / Likely Serious Adverse Effect or Requiring Medical Treatment Form **51**

SYNOPSIS

**Name of sponsor :**	PIERRE FABRE DERMO-COSMÉTIQUE
**Name/Code of product :**	D-Pigment rich texture / RV4280A
**Title of the study :**	Efficacy and tolerance of D-pigment versus moisturizer in hands' lentigo lesions with or without laser therapy.
**Principal Investigator :**	Pr Giovanni PELLACANI
**Study centre:**	Department of Dermatology<br>University of Modena and Reggio Emilia<br>Via del pozzo 71<br>41100 MODENA
**Study rationale :**	A lentigo is a small, sharply circumscribed, pigmented lesion surrounded by normal-appearing skin. Involved sites of solar lentigos frequently include the backs of the hands, face, forearms, back, neck, and chest. There are many types of lentigos, including lentigo simplex and solar or actinic lentigo which are the most frequent and benign lentigos. Lentigos occur as a result of over exposure to the sun. Their incidence, number and size increase with chronological aging and UV exposure.<br><br>Many studies have shown the efficacy of topical products such as hydroquinone, tretinoin, ascorbic and phytic acid... Many physical therapies such as chemical peels, cryosurgery, dermabrasion, and pigment-specific lasers show good results.<br><br>The combination of physical and topical therapies may be beneficial to patients. Laser therapy is very efficient on visible lesion, but not to prevent lesions apparition. Furthermore, Post-inflammatory hyperpigmentation (PIH) is a common adverse effect seen with laser, occurring in approximately 35–40% of patients with Fitzpatrick skin types I–III. A cosmetic topical depigmenting product may be a support of laser therapy, to prevent lentigos apparition in peri-lesional areas and to prevent possible laser side effect.<br><br>Pierre Fabre Dermo-Cosmétique has commercialized a cosmetic product (D-pigment rich texture) for treatment of hyperpigmentation. This product show good results on smoothing irregularities, reducing and preventing dark spots. The aim of this study is to evaluate the efficacy of D-pigment after laser therapy, through its action on peri-lesional areas, in obtaining better cosmetic effect..
**Study Period :**	First half of 2013 to second half 2014

**Objectives of the study :**	**<u>Primary objective:</u>**<br>To assess at 12 months, the efficacy of D-Pigment versus moisturizer by quotation of the homogeneity of the skin colour, by blinded evaluation from standardized photos on the two global hands. The homogeneity of the skin colour is evaluated through colour difference and affected surface quotation on Visual Analogue Scales (VAS).<br><br>**<u>Secondary objectives :</u>**<br><u>Objectives on global areas</u><br>The evolution of the parameter will be assessed in time on each area and the evolution will be compared between the different areas: D-pigment hand versus moisturizer hand, area treated by laser on D-pigment hand versus area treated by laser on moisturizer hand, area not treated by laser on D-pigment hand versus area not treated by laser on moisturizer hand.<br><br>- To asses the homogeneity of the skin colour (evaluated through colour difference and affected surface quotation) on global hands, area treated by laser and area not treated by laser of both hands.<br>- at T0, 3, 6 and 9 months by blinded evaluation from standardized photos<br>- at T0, 3, 6, 9 and 12 months by clinical evaluation<br><br>- To assess the number of lentigos on global hands, area treated by laser and area not treated by laser of both hands, at T0, 3, 6 , 9 and 12 months by clinical evaluation and blinded from standardized photos<br><br><u>Objectives on 'target lesions'</u><br>The evolution of the parameter will be assessed in time on four 'target lesions': a target lesion previous treated by laser and a target lesion not treated by laser on each hand.<br>The evolution will also be compared between the different 'target lesions':<br>- target lesion treated by laser on D-pigment hand versus target lesion treated by laser on moisturizer hand<br>- target lesion not treated by laser on D-pigment hand versus target lesion not treated by laser on moisturizer hand<br>- target lesion treated by laser on D-pigment hand versus target lesion not treated by laser on D-pigment hand<br>- target lesion treated by laser on moisturizer hand versus target lesion not treated by laser on moisturizer hand.<br><br>- To assess the change in chromametry parameters (L*, a*, b*) and the colour homogeneity on the target lesion treated by laser and the target lesion not treated by laser of both hands, at T0, 3, 6 , 9 and 12 months by camera measurement.<br><br>- To assess at the change in confocal microscopy parameters (pigment distribution, epidermal thickness, papillae contours) on the target lesion treated by laser and the target lesion not treated by laser of both hands at T0, 3, 6 , 9 and 12 months<br><br>- To assess the dynamic Physician Global Assessment (PGA) on the target lesion treated by laser and the target lesion not treated by laser of both hands at T0, 3, 6 , 9 and 12 months.<br><br><u>Objectives through subject assessment</u><br>- To assess the overall efficacy assessed by the subjects on global hands, area treated by laser and area not treated by laser of both hands at 3 and 12 months<br><br>- To assess the satisfaction regarding to the use of D-Pigment through a questionnaire<br><br>- To assess the tolerance of D-Pigment at 3, 6, 9 and 12 months.

**Methodology :**	Intra-individual, randomized, hand controlled open label study
**Number of subjects :**	30 subjects must be analysed in the study. Assuming that some subjects will be withdrawn from the study during the 12 months of follow up and others will be not entirely analysable, 40 subjects will be recruited for the study.
**Inclusion Criteria :**	**Related to the population:**<br>- Male or female aged over 50 years,<br>- Phototype II to IV,<br>- Subject consenting on the use of hand-photos for scientific and commercial purposes,<br>- Subject having signed his/her written informed consent.<br><br>**Related to pathology:**<br>- Subject treated by laser therapy in the previous 15 to 30 days<br>- Subject having 5 to 10 lentigos on the surface of each hands<br>- Subject having lentigos graded 6 or more on the severity grading scale<br>- Subject with same lentigo severity in each hand.

**Non Inclusion Criteria :**	**Related to the population:**<br>- Subject who has planned to stay for more than 15 days in an area with an important increase in sun exposure conditions compared to his/her usual place of residence,<br>- Subject who has planned to sun expose himself/herself more than 7 days,<br>- Women with childbearing potential<br>- Participation to an other clinical trial in the previous month or during the study,<br>- Subject who is not able to understand the information (for linguistic or psychiatric reasons), to give informed consent,<br>- Subject who, in the judgement of the investigator, is not likely to be compliant with study-related constraints during the study (daily product application during one year),<br>- Subject who has forfeited her freedom by administrative or legal decision, or who is under guardianship.<br><br>**Related to pathologies:**<br>- Hyper pigmentation other than lentigos or other hypermelanosis (post-inflammatory laser or chemical melanosis) on the hands,<br>- Diabetic subject,<br>- Chronic or progressive disease which may interfere with the study in the opinion of the investigator,<br>- Pathology, skin disorder or lesions other than lentigo (psoriasis, atopic dermatitis, mycose, intertrigo, sunburn…) at the dorsum of the hand which could interfere with the evaluation,<br>- Systemic infectious pathology,<br>- Hypersensitivity, allergy or intolerance to retinaldehyde or any component of the formulation.<br><br>**Related to treatments:**<br>- Treatment by depigmenting cosmetic products on the hands within 4 weeks prior to inclusion,<br>- Treatment by depigmenting treatment containing hydroquinone or any depigmenting drug on the hands (e.g. topical retinoids, topical steroids, …) within 8 weeks prior to inclusion,<br>- Previous treatment by chemical peels, dermabrasions on the hands within one year prior to inclusion,<br>- Treatment by photosensitive agents within 8 weeks prior to inclusion.
**Specific requirements :**	Subjects will not be allowed to use any cutaneous product on the hands the day of the visits (study products or other cutaneous products or treatment).
**Test product :**	Name : D-Pigment rich texture<br>Product Code : RV4280A<br>Mode of administration: one application once a day on the one hand (according to the randomization) on the evening after cleaning. D-pigment must be applied during one year.<br>Application: cutaneous use<br>Maximum duration of application : 360 days ± 15

**Reference product :**	Name : Hydrance optimale riche<br>Product Code : RV2300G<br>Mode of administration: one application once a day on the other hand (according to the randomization) on the evening after cleaning. Hydrance must be applied during one year.<br>Application: cutaneous use<br>Maximum duration of application : 360 days ± 15
**Evaluation Criteria**<br><br>➢ **Efficacy :**	**<u>Primary Criterion</u>:**<br><br>Homogeneity of the skin colour assessed at 12 months by blinded evaluation from standardized photos.<br>Homogeneity of the skin colour assessed is defined through two Visual Analogue Scales. One VAS grades the surface affected by lentigo on the observed area (no lentigo in the area corresponding to 0, area completely hidden by lentigo corresponding to 10). The second VAS grades the difference between lighter and darker skin of the area (same colour of skin on the area corresponding to 0, extremely dark lentigo on the area corresponding to 10). The sum of the two components (0 to 20) represents the homogeneity of the skin colour<br>The evolution between T0 and 12 months of homogeneity of skin colour will be assessed on global D-pigment hand and global moisturizer hand.<br>The evolution after 12 months of product application will be compared between the two hands.<br><br>**<u>Secondary criteria:</u>**<br><br>o **Criteria assessed on global area**<br>Criteria 1 and 2 will be calculated with two different methods: from clinical evaluation during the visit and from blinded evaluation from standardized photos.<br>Criteria 1 and 2 will be compared between the global hands, area treated by laser and area not treated by laser on both hands.<br><br>**1** - Homogeneity of the skin colour assessed at T0, 3, 6 and 9 months (and 12 months for dermatological examination)<br>The skin colour homogeneity will be assessed on each global hand, on area treated by laser and area not treated by laser of each hand.<br><br>**2** - Lentigos number assessed at T0, 3, 6, 9 and 12 months<br>The lentigos will be count on each global hand, on area treated by laser and area not treated by laser of each hand.

➢ **Efficacy :**	o **Criteria assessed on target lesions**<br>Criteria 3, 4, 5 and 6 will be compared between four "target lesions": a target lesion previous treated by laser (and its peri-lesional area) and a target lesion not treated by laser (and its peri-lesional area) on each hand.<br><br>**3 -** Chromametry parameters using camera (L*, a*, b*) at T0, 3, 6, 9 and 12 months<br>Colorimetric evaluations (L*, a* and b*) will be carried out from pictures obtained by camera at T0, 3, 6, 9 and 12 months. A program will calculate colour from camera acquisition.<br><br>**4** - Confocal parameters:, measured by confocal microscopy at T0, 3, 6, 9 and 12 months<br><br>**5** - Dynamic Physician Global Assessment (PGA) assessed through a 7 points scale at T3, 6, 9 and 12 months.<br><br>o **Criteria assessed by the subjects**<br><br>**6 -** Overall efficacy assessed by the Subject through a 5 points scale at 3 and 12 months.<br><br>**7 -** Satisfaction regarding to the use of D-Pigment assessed by the subject through a questionnaire after 3 months of daily applications.
➢ **Tolerance :**	- The tolerance will be assessed by the investigator at 3, 6, 9 and 12 months, on a 4-point grading scale :<br>1 = Very good tolerance: no functional or physical sign from examination<br>2 = Good tolerance: transitory functional signs and no physical signs from examination<br>3 = Poor tolerance: persisting functional signs or physical signs from examination leading to modification of the conditions of administration but no application discontinuation<br>4 = Very poor tolerance: functional and/or physical signs from examination leading to application discontinuation<br><br>- Reporting of adverse events
➢ **Investigational Plan :**	**<u>Product application</u>**<br>The subjects will be recruited after an incomplete laser therapy, performed on the two hands. D-pigment will be applied on one hand and moisturizer will be applied on the other. The site of application will be defined by random.<br>**<u>Conduct of the study</u>**<br>40 subjects will be recruited for the study.<br>At the first visit V1 (D1), the investigator will check all inclusion and non inclusion criteria, then the products application will be explained. From V1 to V5, the products will be applied once a day, during one year.<br>The subject will attend 4 follow-up visits, every 3 months: V2 (D90 ± 8), V3 (D180 ± 8), V4 (D270 ± 15) and V5 (D360 ± 15). During these visits, all the criteria will be checked, particularly dermatological examination, photos will be taken, camera and confocal microscopy measurement will be done.

➢ **Statistical Methods :**	Qualitative parameters will be tabulated by frequencies and percentages by group and visit. Quantitative parameters will be represented by n, missing, mean, std median and range, by group and visit.<br><br>For the primary analysis, the change between T0 and T12 months will be compared between product using an analysis of covariance (ANCOVA) with product, site and sequence as fixed factor, subject as random factor and value at baseline as covariate.<br><br>For other criteria measured at each visit, a MMRM approach will be used.<br><br>For overall efficacy and cosmetic agreement questionnaire, only a descriptive approach will be used (frequencies and percentages).

STUDY FLOW CHART

	Visit 1<br>1 (day 1)<br>Inclusion visit	Visit 2<br>(day 90 ± 8)<br>Intermediate visit	Visit 3<br>(day 180 ± 8)<br>Intermediate visit	Visit 4<br>(day 270 ± 15)<br>Intermediate visit	Visit 5<br>(day 360 ± 15)<br>End of study visit
**Subject information**	✔				
**Written Informed consent**	✔				
**Medical and Surgical History**	✔				
**Concomitant diseases**	✔				
**Previous treatment**	✔				
**Concomitant treatment**	✔	✔	✔	✔	✔
**Hands dermatological examination**	✔	✔	✔	✔	✔
**Dermatologist evaluation of lentigos number**	✔	✔	✔	✔	✔
**Inclusion/ Non inclusion criteria**	✔				
**Report of adverse events**		✔	✔	✔	✔
**Digital Standardized Photos**	✔	✔	✔	✔	✔
**Camera measurement**	✔	✔	✔	✔	✔
**Confocal microscopy measurement**	✔	✔	✔	✔	✔
**Static Physician Global Assessment**	✔				
**Dynamic Physician Global Assessment**		✔	✔	✔	✔
**Overall efficacy assessed by subject**		✔			✔
**Satisfaction regarding to the use of D-Pigment**		✔			
**Tolerance**		✔	✔	✔	✔
**Dispensing of study products and subject diary**	✔	✔	✔	✔	
**Recovery of study products and subject diary**		✔	✔	✔	✔

List of Abbreviations

**AE**	:	Adverse event
**CA**	:	Competent Authority
**CRA**	:	Clinical Research Associate
**CRF**	:	Case Report Form
**CRO**	:	Contract Research Organization
**D**	:	Day
**Dr**	:	Doctor
**EC**	:	Ethics Committee
**FAS**	:	Full Analysis Set
**HA**	:	HydroxyAnisole
**GCP**	:	Good Clinical Practices
**ICH**	:	International Conference on Harmonisation
**IRPF**	:	Institut de Recherche Pierre Fabre
**ITT**	:	Intention To Treat
**PFB**	:	Pierre Fabre Biometrie
**PFDC**	:	Pierre Fabre Dermo-Cosmétique
**PGA**	:	Physician Global Assessment
**PIH**	:	Post-inflammatory hyperpigmentation
**PP**	:	Per Protocol
**RAL**	:	Retinaldehyde
**RAS**	:	Rapport Annuel de Sécurité
**SAE**	:	Serious Adverse Event
**T**	:	Time
**VAS**	:	Visual Analogue Scale
**WHO**	:	World Health Organisation

1. INTRODUCTION AND STUDY RATIONALE

1.1. TARGET PATHOLOGY AND THERAPY

A lentigo is a small, sharply circumscribed, pigmented lesion surrounded by normal-appearing skin. The initial appearance of lentigos varies and depends on the race, the sun exposures, genetic predisposition and other factors, depending on the type of lentigo. Lentigos can range in size from few millimetres up to a few centimetres in diameter and, in areas of severely sun-damaged skin. Involved sites of solar lentigos frequently include the backs of the hands, face, forearms, back, neck, and chest (1).

There are many types of lentigos, including lentigo simplex and solar or actinic lentigo which are the most frequent and benign lentigos. These two types differ on age and sun exposure. Lentigos occur as a result of over exposure to the sun, as the process of aging begins than the skin becomes more and more prone to injury by sun. Their incidence, number and size increase with chronological aging and UV exposure (2).

This is an epidermal hypermelanocytosis. Histologic findings may include hyperplasia of the epidermis and increased pigmentation of the basal layer. There is an increased number of melanocytes in the epidermis leading to an excessive production of melanin in epidermis. (2-3-4-5)

Therapy for solar lentigos can be divided into two broad categories: physical therapy and topical therapy.
A range of topical therapies are currently in use, including hydroquinone, tretinoin, kojic and retinoic acid…
Topical therapies usually achieve a good result but take longer than physical therapies.
Combinations of these compounds (4HA/tretinoin) are used and show efficacy in treating lentigos (6).
Physical therapy of lentigos includes cryotherapy, laser therapy, pulsed light, and chemical peels. These are frequently used with excellent clinical success rates; however, this type of therapy should be balanced against associated side effects and recurrence rates with certain therapies. (7)

1.2. INFORMATION ON THE TEST PRODUCT

There test product is D-pigment rich texture, this cream is marketed in Italy since September 2012.

D-pigment rich texture is a cosmetic non rinsed product used in the treatment of epidermal hyperpigmentation. The cream is used to smooth irregularities, to reduce and prevent dark spots.
The safety of this product has already been investigated. Clinical studies have demonstrated good tolerance of D-Pigment. D-Pigment rich texture is formulated without parabens and fragrance. It associates three ingredients, the Symwhite (Phenyl ethyl resorcinol) 0,5%, the retinaldehyde (RAL) 0,05% and the Tocopheryl glucoside 0,1%.

- **Phenyl ethyl resorcinol** (0,5 %). It is a synthetic compound obtained by modification of 3-5 dihydroxystilbene (consisted stemming from a lipophilic extract of the Scotsh pine: Pinus Sylvestris). The hydroxystilbenes demonstrated a very good action on the pigmentation by the inhibition of the tyrosinase (8-9) key enzyme of the melanin synthesis. A study (10) demonstrated in vitro (on the synthesis of the melanin) and in vivo (on the skin lightening) that Phényl ethyl résorcinol was the most active compound of the series with an activity superior to Kojic acid and to the hydroquinone, reference molecules in term of skin lightening activity.

- **Retinaldehyde** (RAL) 0, 05%. The retinoids are a class of chemical compounds that include the A vitamin (retinol) and its derivatives. Among them, the RAL is the natural precursor of the retinoic acid and is less irritating than this last one. In topical application, the RAL stimulates keratinocytes proliferation and increases the natural cellular turn over of the skin, allowing the faster elimination of the pigmented cells (11).

- **Tocopheryl glucoside**: For this ingredient two effects were put in evidence: an activity of inhibition of the melanogenesis (dose effect depending) demonstrated in vitro on culture of murine B16 melanocytes and a cellular antioxidant and protective effect (12).

1.3. STUDY RATIONALE

Many studies have shown the efficacy of topical products such as hydroquinone, tretinoin, ascorbic and phytic acid... The combination of topical agents has shown better improvement of lentigos aspect (6-13). Many physical therapy such as chemical peels, cryosurgery, dermabrasion, and pigment-specific lasers show good results (7-14).

The combination of physical and topical therapies may be beneficial to patients. For example, the use of combination topical therapy may be used after cryotherapy as a maintenance therapy to diminish the risk of relapse (7). Laser therapy is very efficient on visible lesion, but not to prevent lesions apparition. A cosmetic topical depigmenting product may be a support of laser therapy, to prevent lentigos apparition in peri-lesional areas.

Furthermore, Post-inflammatory hyperpigmentation (PIH) is a common adverse effect seen with laser, occurring in approximately 35–40% of patients with Fitzpatrick skin types I–III (15). Another study related that 28% of study population has post-inflammatory hyperpigmentation (14). A cosmetic topical depigmenting product may be a support of laser therapy, to prevent possible laser side effect.

Pierre Fabre Dermo-Cosmétique has commercialized a cosmetic product (D-pigment rich texture) for treatment of hyperpigmentation. This product show good results on smoothing irregularities, reducing and preventing dark spots. The aim of this study is to evaluate the efficacy of D-pigment as a support of a laser therapy, through its action on peri-lesional areas.

1.4. OVERALL RISK AND BENEFIT ASSESSMENT

The expected effect is to show the efficacy of D-pigment in post laser long-term treatment, having a more homogenous skin colour on the D-pigment hand versus the moisturizer hand. The subjects will benefit from a strengthened follow-up by the investigator (one visit every 3 months). After the completion of the study, a complete hands laser treatment will be done for each subject and D-pigment tubes will be offered to the subjects for a 6 months application period.

Some risks related to the products may exist. Depending on the skin of person, there may be a risk of local irritation, such as skin reddening, peeling, mild burning sensation, dryness, and pruritus at the site of application. The safety of the products will be assessed by the investigator at Visit 2, Visit 3, Visit 4 and Visit 5. In case of suspected allergic reaction to one of the products, the investigator will propose an allergic test to the different ingredients of the product. The subject will be monitored by the investigator throughout the study.

The instrumental assessments (confocal microscopy, camera measurements and standardized photos) are noninvasive and painless.

2. STUDY OBJECTIVES

2.1. PRIMARY OBJECTIVE

To assess at 12 months, the efficacy of D-Pigment versus moisturizer by quotation of the homogeneity of the skin colour, by blinded evaluation from standardized photos on the two global hands. The homogeneity of the skin colour is evaluated through colour difference and affected surface quotation on Visual Analogue Scales (VAS).

2.2. SECONDARY OBJECTIVES

Objectives on global areas
The evolution of the parameter will be assessed in time on each area and the evolution will be compared between the different areas: D-pigment hand versus moisturizer hand, area treated by laser on D-pigment hand versus area treated by laser on moisturizer hand, area not treated by laser on D-pigment hand versus area not treated by laser on moisturizer hand.

- To asses the homogeneity of the skin colour (evaluated through colour difference and affected surface quotation) on global hands, area treated by laser and area not treated by laser of both hands.
  - at T0, 3, 6 and 9 months by blinded evaluation from standardized photos
  - at T0, 3, 6, 9 and 12 months by clinical evaluation

- To assess the number of lentigos on global hands, area treated by laser and area not treated by laser of both hands, at T0, 3, 6 , 9 and 12 months by clinical evaluation and blinded from standardized photos

Objectives on 'target lesions'

The evolution of the parameter will be assessed in time on four 'target lesions': a target lesion previous treated by laser and a target lesion not treated by laser on each hand.
The evolution will also be compared between the different 'target lesions':

- target lesion treated by laser on D-pigment hand versus target lesion treated by laser on moisturizer hand
- target lesion not treated by laser on D-pigment hand versus target lesion not treated by laser on moisturizer hand
- target lesion treated by laser on D-pigment hand versus target lesion not treated by laser on D-pigment hand
- target lesion treated by laser on moisturizer hand versus target lesion not treated by laser on moisturizer hand.

- To assess the change in chromametry parameters (L*, a*, b*) and the colour homogeneity on the target lesion treated by laser and the target lesion not treated by laser of both hands, at T0, 3, 6 , 9 and 12 months by camera measurement.

- To assess at the change in confocal microscopy parameters (pigment distribution, epidermal thickness, papillae contours) on the target lesion treated by laser and the target lesion not treated by laser of both hands at T0, 3, 6 , 9 and 12 months

- To assess the dynamic Physician Global Assessment (PGA) on the target lesion treated by laser and the target lesion not treated by laser of both hands at T0, 3, 6 , 9 and 12 months.

Objectives through subject assessment

- To assess the overall efficacy assessed by the subjects on global hands, area treated by laser and area not treated by laser of both hands at 3 and 12 months

- To assess the satisfaction regarding to the use of D-Pigment through a questionnaire

- To assess the tolerance of D-Pigment at 3, 6, 9 and 12 months

3. ETHICAL CONSIDERATIONS RELATING TO THE STUDY

The trial is conducted according to Good Clinical Practices (CPMP/ICH/135/95) and the Declaration of Helsinki and its subsequent amendments (see Appendix 17.1) and national regulations.

This protocol and related documents (included the informed consent form) will be submitted for approval to independent Ethics Committees before the study set up, according to national regulations.

After being informed orally and in writing of all aspects and constraints of the trial, as well as of their freedom to withdraw their participation at any time, the subjects will then sign a consent on a triplicate form, after a sufficient time of reflection: one copy for the investigator, one for the subject, and one for the sponsor (into a sealed envelope).

A time of reflection will be given to the subjects, before giving a written consent and starting the study procedures.

The subjects will undergo a regular clinical follow-up during the study.

To analyse the effect on D-pigment in hands' lentigo lesions with or without laser therapy, a comparative hand is necessary. Furthermore, to avoid inter-individual variation (bias relating to sun exposition), the intra-individual comparison is justified. At the end of the study both hands will be treated by laser if needed, and D-pigment product will be offered for a 6 months application period.

As safety for use during pregnancy and childhood has not been established for the test product, women of childbearing potential will be excluded from clinical trials.

4. STUDY DESIGN

4.1. OVERALL DESCRIPTION

This study will be conducted as an European, single-centre, randomised, open-label, reference product controlled, intra-individual hand controlled trial, on 40 subjects suffering from hands' lentigos.

5 visits are planned:

- Visit 1 : Inclusion (D1)
- Visit 2 : Intermediate visit (D90 ± 8)
- Visit 3 : Intermediate visit (D180 ± 8)
- Visit 4 : Intermediate visit (D270 ± 15)
- Visit 5 : End of study visit (D360 ± 15)

The duration of participation for a subject is 360 days (± 15).

The subjects will be recruited after a laser therapy, performed in the previous 15 to 30 days on the two hands. The subjects will be recruited if they still have 5 to 10 lentigos (graded 6 or more on the severity grading scale) on the surface of each hands.

D-pigment will be applied on one hand and moisturizer will be applied on the other. The site of application will be defined by random.

At the first visit V1 (D1), the investigator will check all inclusion and non inclusion criteria, then the products application will be explained. From V1 to V5, the products will be applied once a day, during one year.

The subject will attend 4 follow-up visits, every 3 months: V2 (D90 ± 8), V3 (D180 ± 8), V4 (D270 ± 15) and V5 (D360 ± 15). During these visits, all the criteria will be checked, particularly dermatological examination, photos will be taken, camera and confocal microscopy measurement will be done.

4.2. DISCUSSION OF THE STUDY DESIGN

An intra-individual hand controlled trial (left/ right comparison) allows avoiding potential bias relative to the UV exposures, the major factor of apparition and worsening of lentigos. It is also the best methodology for the comparison of the test product versus a reference product.

This study should prove an efficacy of D-pigment in post laser long-term treatment. A follow-up during one year will allow to follow the evolution of lentigos in area treated by laser and peri-lesional area.

Apparition of lentigo is correlated to age and sun exposure. Thus, to analyse lentigo, an ageing population is necessary.

5. STUDY POPULATION

5.1. INCLUSION CRITERIA

Subjects presenting with the following criteria may be included:

Criteria related to the population:

- Male or female aged over 50 years,
- Phototype II to IV,
- Subject consenting on the use of photos for scientific and commercial purposes,
- Subject having signed his/her written informed consent.

Criteria related to pathologies:

- Subject treated by laser therapy in the previous 15 to 30 days
- Subject having 5 to 10 lentigos on the surface of each hands
- Subject having lentigos graded 6 or more on the severity grading scale
- Subject with same lentigo severity in each hand.

The severity is defined by the following 9 points grading scale:

0: Extremely lighter than pigment of surrounding skin (completely depigmented)
1: markedly lighter than pigment of surrounding skin
2: moderately lighter than pigment of surrounding skin
3: slightly lighter than pigment of surrounding skin
4: equal with pigment of surrounding skin
5: slightly darker than pigment of surrounding skin
6: moderately darker than pigment of surrounding skin
7: markedly darker than pigment of surrounding skin
8: extremely darker than pigment of surrounding skin

5.2. NON INLUSION CRITERIA

Subjects presenting with any of the following criteria will not be included:

Criteria related to the population:

- Subject who has planned to stay for more than 15 days in an area with an important increase in sun exposure conditions compared to his/her usual place of residence,
- Subject who has planned to sun expose himself/herself more than 7 days,
- Women with childbearing potential
- Participation to an other clinical trial in the previous month or during the study,
- Subject who is not able to understand the information (for linguistic or psychiatric reasons), to give informed consent,
- Subject who, in the judgement of the investigator, is not likely to be compliant with study-related constraints during the study (daily product application during one year),
- Subject who has forfeited her freedom by administrative or legal decision, or who is under guardianship.

Criteria related to pathologies:

- Hyperpigmentation other than lentigos or other hypermelanosis (post-inflammatory laser or chemical melanosis) on the hands,
- Diabetic subject,
- Chronic or progressive disease which may interfere with the study in the opinion of the investigator,
- Pathology, skin disorder or lesions other than lentigo (psoriasis, atopic dermatitis, mycose, intertrigo, sunburn…) at the dorsum of the hand which could interfere with the evaluation,
- Systemic infectious pathology,
- Hypersensitivity, allergy or intolerance to retinaldehyde or any component of the formulation.

Criteria related to treatments:

- Treatment by depigmenting cosmetic products on the hands within 4 weeks prior to inclusion,
- Treatment by depigmenting treatment containing hydroquinone or any depigmenting drug on the hands (e.g. topical retinoids, topical steroids, …) within 8 weeks prior to inclusion,
- Previous treatment by chemical peels, dermabrasions on the hands within one year prior to inclusion,
- Treatment by photosensitive agents within 8 weeks prior to inclusion.

5.3. SPECIFIC REQUIREMENTS

Subjects will not be allowed to use any cutaneous product on the hands the day of the visits (study products or other cutaneous products or treatment).

5.4. NUMBER OF SUBJECTS

30 subjects must be analysed in the study. Assuming that some subjects will be withdrawn from the study during the 12 months of follow up and others will be not entirely analysable, 40 subjects will be recruited for the study.

5.5. RECRUITMENT MODALITIES

Subjects will be enrolled by the investigator.

If necessary, a local announcement will be done, after the CA/EC will authorize the text. Will be mentioned the main characteristics of the subjects (age, pathology) and the phone number to contact.

5.6. SUBJECT IDENTIFICATION

The subjects will be identified by the subject code consisting of :

- Number of the center (2 digits),
- Number of the subject in the centre, by chronological order of inclusion (2 digits).

5.7. WITHDRAWAL CRITERIA

The reasons for a subject's premature withdrawal from the study may be the following :

- The subject's decision. A subject who wishes to withdraw from the study for any reason may do so at any time but must inform the investigator. In all cases, the investigator should attempt to contact the subject as soon as possible for a final assessment in order to :
  - have the subject's decision written on the consent form
  - obtain the reason(s) for withdrawal and report it/them in the case report form,
  - evaluate the subject's clinical condition,
  - if necessary, take appropriate therapeutic measures: management of an adverse effect or concomitant disease, prescription of another treatment.
- The investigator's decision in the subject's interest. The monitor will be informed by phone or fax and a letter or report explaining the withdrawal will be forwarded to the monitor as soon as possible.
- An erroneous inclusion according to the protocol. The decision to maintain or not the subject in the study will be taken jointly by the investigator and the sponsor.
- Other reasons (insufficient response...)

In all cases, available data will be retained for the safety analysis.

5.8. REPLACEMENT OF SUBJETS

Subjects prematurely withdrawn or lost to follow-up will not be replaced.

5.9. POST-STUDY EXCLUSION PERIOD

The subjects will not be allowed to participate to an other clinical trial during the overall study period and during 1 month following the study termination.

6. STUDY PRODUCTS

6.1. SOURCE, PRESENTATION AND COMPOSITION OF INVESTIGATIONAL PRODUCTS AND ASSOCIATED PRODUCT

6.1.1. Test product

Name : D-Pigment rich texture
Product Code : RV4280A
Formulation Code : ND34000
Presentation : topical cream
Composition : WATER (AQUA), CAPRYLIC/CAPRIC TRIGLYCERIDE, OCTYLDODECANOL, AVENE THERMAL SPRING WATER (AVENE AQUA), CETEARYL ALCOHOL, GLYCERIN, BEHENYL ALCOHOL, AMMONIUM ACRYLOYL DIMETHYLTAURATE/VP COPOLYMER, CETEARETH-33, DIPENTAERYTHRITYL PENTAISONONANOATE, BENZOIC ACID, BHT, DISODIUM EDTA, OENOTHERA BIENNIS (EVENING PRIMROSE) OIL (OENOTHERA BIENNIS OIL), PHENOXYETHANOL, PHENYLETHYL RESORCINOL, RED 33 (CI 17200), RETINAL, SILICA, SODIUM HYDROXIDE, TOCOPHEROL, TOCOPHERYL GLUCOSIDE.

6.1.2. Reference Product

Name : Hydrance optimale riche
Product Code : RV2300G
Formulation Code : VM0902
Presentation : topical cream
Composition : AVENE THERMAL SPRING WATER (AVENE AQUA), MINERAL OIL (PARAFFINUM LIQUIDUM), GLYCERIN, PPG-15 STEARYL ETHER, CYCLOMETHICONE, CETEARYL ALCOHOL, CARTHAMUS TINCTORIUS (SAFFLOWER) SEED OIL (CARTHAMUS TINCTORIUS SEED OIL), GLYCERYL STEARATE, PEG-100 STEARATE, BUTYROSPERMUM PARKII (SHEA BUTTER) (BUTYROSPERMUM PARKII BUTTER), CETEARYL GLUCOSIDE, BENZOIC ACID, BETA-SITOSTEROL, BHT, C13-14 ISOPARAFFIN, CAPRYLIC/CAPRIC TRIGLYCERIDE, CHLORPHENESIN, DISODIUM EDTA, FRAGRANCE (PARFUM), GLYCINE SOJA (SOYBEAN) SEED EXTRACT (GLYCINE SOJA SEED EXTRACT), HYDROGENATED COCO-GLYCERIDES, LAURETH-7, LECITHIN, PEG-32, PEG-400, PHENOXYETHANOL, POLOXAMER 188, POLYACRYLAMIDE, SODIUM CHONDROITIN SULFATE, SODIUM HYDROXIDE, WATER (AQUA), XANTHAN GUM.

6.2. PACKAGING AND LABELLING

The labelling of the study products will be conducted by the monitor of the study.

6.2.1. Packaging

Primary packaging: The study products will be packaged in commercial tubes of 30 ml for the test product and 40 ml for the reference product.

Secondary packaging: two tubes of the test product and the reference product will be grouped together in a paper bag labelled with the study code and subject number. This secondary packaging shall constitute the study kit for three months.

6.2.2. Labeling

Labelling should comply with the local requirements.

The following information should be included on labels:

- Name and address of the Sponsor
- Study Code : RV4280A2012607
- Subject Number
- Right hand / left hand
- Expiry date
- Statements « External Use », « Comply with the instructions for use »
- Statements : « For clinical use only »

6.3. DISTRIBUTION TO CENTRE AND STORAGE

The monitor will supply the necessary study products to the Investigational centre before the study start.

As soon as the Investigator receives the investigational products, he/she will check the content and immediately return to the monitor one of the two letters of acknowledgment of receipt that accompany the parcel, duly completed and signed. The second letter will also be completed and kept in the Investigator's file. The same procedure will be applied to all further supplies during the course of the trial.

The CRA will ensure during the initiation visit that study products have been received in good conditions and the acknowledgement of receipt has been returned adequately.

The study products should remain intact until dispensation to the subjects. They should be kept without specific instruction for storage, in a appropriate lockable room only accessible to the person authorised to dispense them, under the responsibility of the pharmacist or the investigator.

6.4. ALLOCATION OF PRODUCTS AND DISPENSATION TO SUBJECTS

A randomisation list will be established by Pierre Fabre Biometrie. This department will keep the randomisation table until the end of the study (statistical analysis).

At the randomization visit (Visit 1 at Day 1), the Investigator will have to allocate the study products to each subject according to the chronological order of arrival at the randomization visit, in order to respect the randomization previously established.

The investigator will only dispense the study products to the subjects included in the study.

The study products will be dispensed to the subjects by the investigator at the following visits: Visit 1 (D1), Visit 2 (D90± 8), Visit 3 (D180 ± 8) and Visit 4 (D270 ± 15).

6.5. PRODUCTS ADMINISTRATION

6.5.1. Duration of products application

For a subject completing the study, the maximal theoretical study product exposure will be 375 days (360 days + 15 days).

6.5.2. Route, dosage and conditions of administration

The study products will be applied once daily on the dorsum of the hand (a product fingertip on each hand) the evening after cleaning. The test product will be applied on one hand and the reference product on the other hand, according to the randomization.

6.6. ACCOUNTABILITY ON SITE AND RETURN/DESTRUCTION OF INVESTIGATIONAL PRODUCTS AND ASSOCIATED PRODUCT

The Investigator should maintain accurate written records of investigational products received, dispensed, returned and any remaining study products, on the product accountability form. These records should be made available for Clinical Research Associate (CRA) monitoring. The packaging of the study products should remain intact until just before the product delivery

At the end of the study, all used and unused products including packaging should be noted and the return to the monitor will be organised by the CRA for destruction.

A copy of the product accountability form should be provided by the Investigator to the CRA

6.7. RECALL OF INVESTIGATIONAL PRODUCTS

In case of recall of investigational products (decided by the Competent Authorities or the sponsor), the investigator will be immediately informed by the sponsor.

The investigator, in collaboration with the sponsor representatives (monitor, CRA) must urgently :

- stop the delivery of the concerned investigational products to the subjects
- inform the concerned subjects that they must immediately stop taking these investigational products and bring them back.

The monitor/CRA organises the return of the recalled products according to the sponsor procedures.

7. CONCOMITANT TREATMENTS

Any existing concomitant treatment or products used on the hands, any new concomitant treatment or change in the dosage of an existing concomitant treatment or products used on the hands during the course of the study must be noted in the relevant section of the CRF.

7.1. PROHIBITED TREATMENTS, PRODUCTS AND PARTICULAR PRACTICE CONCERNING THE SKIN

Depigmenting treatment and depigmenting cosmetic products on the hands,
Any potentially photosensitive formulations or drugs,
Abrasive cosmetic products on the hands,
Cosmetic products with alcohol on the hands,
Any other treatment liable to interfere with the course and/or assessment of the study indication,
Chemical peels, laser treatments, dermabrasions on the hands,
Sunlamp and Sun exposures.

7.2. AUTHORISED TREATMENTS, PRODUCTS AND PARTICULAR PRACTICE CONCERNING THE SKIN

All treatments, products and particular practice concerning the skin other than those prohibited are authorized.
All other treatments, products applied on the hands and particular practice concerning the skin must be clearly documented. The investigator will carefully investigate for any other treatments taken, products applied on the hands and particular practice concerning the skin made by the subject during the study in the course of each of the visits. All treatments received, products applied on the hands and particular practice concerning the skin made by the subject will be accurately described in the case report form (name, form, dosage, route and dosing frequency, treatment initiation, etc.).

8. EVALUATION CRITERIA

8.1. EFFICACY ASSESSMENT

8.1.1. Primary Criterion

Homogeneity of the skin colour assessed at **12 months** by **blinded evaluation from standardized photos.**

The evolution between T0 and 12 months of homogeneity of skin colour will be assessed on global D-pigment hand and global moisturizer hand.
The evolution after 12 months of product application will be compared between the two hands.

Scoring homogeneity
Homogeneity of skin colour will be assessed through 2 Visual Analogue Scales.
One VAS grades the surface affected by lentigo on the observed area, with no lentigo in the area corresponding to 0 and the area completely hidden by lentigo corresponding to 10.
The second VAS grades the difference between the lighter and the darker skin of the area, with the same colour skin on the area corresponding to 0 and extremely dark lentigo corresponding to 10.
The sum of the two components (0 to 20) represents the homogeneity of the skin colour

Blinded evaluation
During all the visits, a person (the investigator or another person under his/her responsibility) will take photos. After the visits, these photos will be renamed and resampled. The person who will evaluate the parameter may be independent from the person who took photos or the same person but the renaming and the resampling will ensure a blinded evaluation.
Each parameter will be evaluated by the same person avoiding thus quotation bias. The parameters will be evaluated on each area blinded from product applied and from time evaluated.
The appraiser will quote at the same time the pictures obtained for one subject, for one hand, at the different times evaluated for the study (T0, T3, T6, T9 and T12 months).

8.1.2. Secondary criteria

8.1.2.1. Criteria assessed on global areas

Criteria 1 and 2 will be calculated with two different methods.
For the first method, the parameters will be obtained from clinical evaluation during the visit. The investigator will grade parameters examining the subject skin and reporting parameters on the CRF.
For the second method, a blinded evaluation from standardized photos as described hereinabove (8.1.1) will be done.

For the criteria 1 and 2, the parameters will be compared as describes hereunder:

The evolution of the parameter will be assessed in time on each area:

- global D-pigment hand
- area treated by laser on D-pigment hand
- area not treated by laser on D-pigment hand
- global moisturizer hand
- area treated by laser on moisturizer hand
- area not treated by laser on moisturizer hand

The evolution at 3, 6, 9 and 12 months will be compared between:

- D-pigment hand versus moisturizer hand
- area treated by laser on D-pigment hand versus area treated by laser on moisturizer hand
- area not treated by laser on D-pigment hand versus area not treated by laser on moisturizer hand

1 - Homogeneity of the skin colour assessed at **T0, 3, 6, 9 and 12 months**
The evaluation of skin colour homogeneity will be done as described hereinabove (8.1.1).
The skin colour homogeneity will be assessed on each global hand, on area treated by laser and area not treated by laser of each hand.

2 - Lentigos number assessed at **T0, 3, 6, 9 and 12 months**

The lentigos will be count on each global hand, on area treated by laser and area not treated by laser of each hand.

8.1.2.2. Criteria assessed on target lesions

For the following parameters, four "target lesions" will be selected for the analysis: a target lesion previous treated by laser and a target lesion not treated by laser on each hand. The instrument's field of view will allow exploring parameters on the target lesions and the peri-lesional area.
To monitor correctly these target lesions, a positioning mask will be prepared at the inclusion visit which will be used during each visit.

For the criteria 3, 4 and 5, the parameters will be compared as describes hereunder:

The evolution of the parameters will be assessed in time on :

- target lesion treated by laser on D-pigment hand
- target lesion not treated by laser on D-pigment hand
- target lesion treated by laser on moisturizer hand
- target lesion not treated by laser on moisturizer hand

The evolution of the parameters at 3, 6, 9 and 12 months of product application will be compared between:

- target lesion treated by laser on D-pigment hand versus target lesion treated by laser on moisturizer hand
- target lesion not treated by laser on D-pigment hand versus target lesion not treated by laser on moisturizer hand
- target lesion treated by laser on D-pigment hand versus target lesion not treated by laser on D-pigment hand
- target lesion treated by laser on moisturizer hand versus target lesion not treated by laser on moisturizer hand

3 - Chromametry parameters using camera (L*, a*, b*) at **T0, 3, 6, 9 and 12 months**

Colorimetric evaluations (L*, a* and b*) will be carried out from pictures obtained by camera at T0, 3, 6, 9 and 12 months. A program will calculate colour from camera acquisition.
The chromametry parameters will be assessed on the target lesion treated by laser and the target lesion not treated by laser of each hand. Comparisons will also be made between chromametry parameters on the target lesions versus peri-lesional area of each hand.

4 – Confocal parameters at **T0, 3, 6, 9 and 12 months**

The confocal parameters: pigment distribution, epidermal thickness, papillae contours will be obtained through confocal microscopy.

- Pigment distribution: as evaluated as extent of mottled pigmentation by exploring intraepidermal layer (at granulosum and spinosum). Pigment distribution will be quoted with ranks (0= absent; 1=<10%; 2=10-30%; 3=>30%).
- Epidermal thickness: measured from the surface (stratum corneum) up to the first appearance of the dermis within the image and up to the last picture containing epidermis (excluded the area around the hair follicles), The thickness will be expressed in microns.
- Papillae contours: evaluated as “regular round-oval with rimmed contours”=0; “irregular, with scalloped borders”=1; “absence-skin flattening”=2
- Polycyclic papillary contours: corresponding to anastomosing cords of epidermis and/or bulbous projections at the junction, corresponding to epidermal hyperplasia, typical for solar lentigo. Papillary contours will be quoted with ranks (0= absent; 1=<10%; 2=10-30%; 3=>30%).
- Enlarged interpapillary spaces and cystic inclusions: corresponding to acanthosis and milia-like cysts, respectively, characteristic for seborrheic keratosis. Interpapillary spaces and cystic inclusions will be quoted with ranks (0= absent; 1=<10%; 2=10-30%; 3=>30%).

The confocal parameters will be assessed on the target lesion treated by laser and the target lesion not treated by laser of each hand.

5 – Dynamic Physician Global Assessment (PGA) at **T3, 6, 9 and 12 months**

The dynamic PGA is defined through a 7 points scale of the change in severity of pigmentation following product application compared to T0:

0 = completely cleared of undesired pigment; no evidence of cosmetic deficit remaining; 100% improvement
1 = very significant clearance of undesired pigment; minimal evidence of cosmetic deficit remaining; about 90% improvement
2 = significant clearance of undesired pigment; slight evidence of cosmetic deficit remaining; about 75% improvement
3 = moderate clearance of undesired pigment; moderate evidence of cosmetic deficit remaining; about 50% improvement
4 = slight clearance of undesired pigment; marked evidence of cosmetic deficit remaining; about 25% improvement
5 = no change in cosmetic appearance from baseline
6 = cosmetic appearance worse than at baseline

At T0, the severity of pigmentation is measured by the Static PGA. The Static PGA clinical score is a 9 points grading scale of lentigo severity:

0: Extremely lighter than pigment of surrounding skin (completely depigmented)
1: markedly lighter than pigment of surrounding skin
2: moderately lighter than pigment of surrounding skin
3: slightly lighter than pigment of surrounding skin
4: equal with pigment of surrounding skin
5: slightly darker than pigment of surrounding skin
6: moderately darker than pigment of surrounding skin
7: markedly darker than pigment of surrounding skin
8: extremely darker than pigment of surrounding skin

The dynamic PGA will be done on the target lesion and peri-lesional target lesion treated by laser and the target lesion and peri-lesional target lesion not treated by laser of both hands.

The evolution of the dynamic PGA at 3, 6, 9 and 12 months of product application will be compared between:

- target lesion treated by laser on D-pigment hand versus target lesion treated by laser on moisturizer hand
- target lesion not treated by laser on D-pigment hand versus target lesion not treated by laser on moisturizer hand
- target lesion treated by laser on D-pigment hand versus target lesion not treated by laser on D-pigment hand
- target lesion treated by laser on moisturizer hand versus target lesion not treated by laser on moisturizer hand

8.1.2.3. Criteria assessed by the subjects

6 - **Overall efficacy** assessed by the **Subject** at **3 and 12 months**

The overall efficacy is defined through a 5 points scale.

0 = completely improved
1 = mostly improved
2 = slightly improved
3 = no improvement
4 = worse

The subjects will assess the overall efficacy on each global hand, on area treated by laser and area not treated by laser of each hand.
The efficacy assessed by the subjects will be compared at 3 and 12 months of product application between :

- D-pigment hand versus moisturizer hand
- area treated by laser on D-pigment hand versus area treated by laser on moisturizer hand
- area not treated by laser on D-pigment versus area not treated by laser on moisturizer hand

7 - Satisfaction regarding to the use of **D-Pigment**

The satisfaction regarding to the use of D-pigment (conditions of use, organoleptic properties, effects...) will be assessed by the subject through a questionnaire after 3 months of daily applications.

8.2. SAFETY ASSESSMENT

8.2.1. Adverse Events

At inclusion, any concomitant disease will be reported on the CRF. At each further visit, the occurrence of adverse events (AEs) since the last visit will be determined by the subject's spontaneous reporting, the investigator's non-leading questioning and his/her clinical evaluation. All AEs will be reported on the CRF (cf section 10).

8.2.2. Local tolerance

Local tolerance will be assessed by the investigator at V2, V3, V4 and V5 using the following 4-point grading scale :

1 = Very good tolerance : no functional or physical sign from examination
2 = Good tolerance : transitory functional signs and no physical signs from examination
3 = Poor tolerance : persisting functional signs or physical signs from examination leading to modification of the conditions of administration but no products application discontinuation
4 = Very poor tolerance : functional and/or physical signs from examination leading to products application discontinuation

8.3. Compliance

The compliance will be check through the subject diary. The subjects will be given a subject diary explaining the products applications, reminding the specific requirements. Subjects will have to note any omissions or changes in frequency of the products application. They will also note if they take any treatment or products on the hands or observe any skin signs occurring between the visits. They will have to give it to the investigator at V2, V3, V4 and V5.
Any change in application frequency or mistake in the application of the study products will be reported in the case report form by the investigator.

9. STUDY PROCEDURES

9.1. Testing conditions

During the study, 5 visits are planned: 1 visit for inclusion (V1 at D1), and 4 visits every 3 months (V2 at D90±8, V3 at D180±8, V4 at D270±15 and V5 at D360±15).

The days of the visits, the subjects have to not apply any cutaneous product on the hands (study products or other cutaneous products or treatment).

9.2. Visit 1 – Inclusion visit (Day 1)

During this first visit, the subject will be informed about the purpose, the conduct of the trial, its constraints and potential risks. If he/she agrees, the subject and the investigator will sign the informed consent form in triplicate.

The investigator will ask the subject about his/her medical and surgical history, concomitant disease, previous and concomitant treatment and note his demographics data (age, sex, phototype).

The investigator will ask the childbearing potential of women.

The investigator will do a dermatological examination of the hands, count the lentigos number and determine the lentigos severity.

The investigator will check the criteria of inclusion and non inclusion.

If the subject is included the investigator will do :

- Basal Digital standardized photos of the hands
- Basal camera measurement on the hands
- Basal confocal microscopy measurement on the hands
- Static Physican Global Assessment

Then the investigator will dispense the first subject diary. In this diary, there is study products use conditions, treatment received or product applied on the hands and particular practice done concerning the skin. The subject has to note any omissions or changes in frequency of the products applications. He/she will also note if he/she observes any skin signs occurring between the visits. This subject diary must be returned to the investigator at the next visit.

The investigator will give the first pack of products for the first period: 2 tubes of test product and 2 tubes of reference product.

The specific requirements of the study will be remembered and next appointments will be set.

9.3. VISIT 2 – INTERMEDIATE VISIT (DAY 90 ± 8 DAYS)

The investigator will retrieve the first subject diary and ask the subject about any skin signs occurred on his/her skin since the previous visit. He will recover all the products given at the previous visit, even tubes are empty.

The investigator will ask the subject about his/her concomitant treatment, he will do a dermatological examination of the hands, count the lentigos number and assess the product efficacy on a target lesion.

The investigator will do:
- Digital standardized photos of the hands
- Camera measurement on the hands
- Confocal microscopy measurement on the hands
- Dynamic Physican Global Assessment

The subject will assess the overall efficacy of the product trough a 5 points grading scale.

The subject will complete a questionnaire to assess his/her satisfaction as regard to the use of the test product.

The tolerance of the products will be assessed by the investigator trough a 4 points grading scale.

Then the investigator will dispense the second subject diary and the second pack of products for the next period. The subject diary and all the products must be returned to the investigator at the next visit.

The specific requirements will be remembered and the next appointment will be set.

9.4. VISIT 3 – INTERMEDIATE VISIT (DAY 180 ± 8 DAYS)

The investigator will retrieve the second subject diary and ask the subject about any skin signs occurred on his/her skin since the previous visit. He will recover all the products given at the previous visit, even tubes are empty.

The investigator will ask the subject about his/her concomitant treatment, he will do a dermatological examination of the hands, count the lentigos number and assess the product efficacy on a target lesion.

The investigator will do:
- Digital standardized photos of the hands
- Camera measurement on the hands
- Confocal microscopy measurement on the hands
- Dynamic Physican Global Assessment

The tolerance of the products will be assessed by the investigator trough a 4 points grading scale.

Then the investigator will dispense the third subject diary and the third pack of products for the next period. The subject diary and all the products must be returned to the investigator at the next visit.

The specific requirements will be remembered and the next appointment will be set.

9.5. VISIT 4 – INTERMEDIATE VISIT (DAY 270 ± 15 DAYS)

The investigator will retrieve the third subject diary and ask the subject about any skin signs occurred on his/her skin since the previous visit. He will recover all the products given at the previous visit, even tubes are empty.

The investigator will ask the subject about his/her concomitant treatment, he will do a dermatological examination of the hands, count the lentigos number and assess the product efficacy on a target lesion.

The investigator will do:
- Digital standardized photos of the hands
- Camera measurement on the hands
- Confocal microscopy measurement on the hands
- Dynamic Physican Global Assessment

The tolerance of the products will be assessed by the investigator trough a 4 points grading scale.

Then the investigator will dispense the fourth subject diary and the fourth pack of products for the next period. The subject diary and all the products must be returned to the investigator at the next visit.

The specific requirements will be remembered and the next appointment will be set.

9.6. VISIT 5 – END-OF-STUDY VISIT (DAY 360 ± 15 DAYS)

The investigator will retrieve the fourth subject diary and ask the subject about any skin signs occurred on his/her skin since the previous visit. He will recover all the products given at the previous visit, even tubes are empty.

The investigator will ask the subject about his/her concomitant treatment, he will do a dermatological examination of the hands, count the lentigos number and assess the product efficacy on a target lesion.

The investigator will do:
- Digital standardized photos of the hands
- Camera measurement on the hands
- Confocal microscopy measurement on the hands
- Dynamic Physican Global Assessment

The subject will assess the overall efficacy of the product trough a 5 points grading scale. The tolerance of the products will be assessed by the investigator trough a 4 points grading scale.

10. ADVERSE EVENTS : DEFINITION AND REPORTING

10.1. ADVERSE EVENTS

10.1.1. Definition

An Adverse Event (AE) is defined as any noxious symptom occurring in a subject taking part in biomedical research, whether or not this symptom is related to the research or to studied products.

10.1.2. Grading of Adverse Events

Adverse events are graded as follows:
- Mild : Awareness of signs and symptoms, but easily tolerated
- Moderate : Uncomfortable enough to cause interference with usual activity
- Severe : Incapacity with inability to work or do usual activity

10.1.3. Reporting of Adverse Events

All Adverse Events occurring during the study must be recorded in the CRF with the following information: nature (diagnosis, signs and symptoms), date of onset, date of end, severity, outcome, actions taken, and relationship to study products (tested cosmetic product or reference product) in the investigator's opinion. It must be specified whether the event is serious or not and if required treatment.

All the Adverse Events for which the investigator or the sponsor considers that a relation of causality with one the study products can be reasonably envisaged are considered as suspicions of adverse effects.

10.2. SERIOUS ADVERSE EVENTS (SAE)

10.2.1. Définition

A Serious Adverse Event (SAE) includes but is not necessarily restricted to any event which:

- results in death (whatever may be the cause),
- is life-threatening,
- requires the subject's hospitalisation* or prolongation of current hospitalisation*,
- results in persistent or significant disability/incapacity,
- is a congenital anomaly or birth defect.

Any pregnancy occuring during / after exposure to the study products should be reported on the "Serious Adverse Event (SAE) / Pregnancy / Likely Serious Adverse Effect or Requiring Medical Treatment Form" (see section 10.6)

* any hospitalisation, or prolongation of hospitalisation due to the circumstances listed below will not be reported as a SAE :

- planned (as per protocol) medical/surgical procedure,
- preparation for routine health assessment/procedure (e.g. routine colonoscopy),
- planned medical/surgical admission (planned prior to entry into study trial, appropriate documentation required),
- administrative or social reasons (e.g. lack of housing, economic inadequacy, family circumstances)

10.2.2. Reporting of SAE

All Serious Adverse Events, according to the above-mentioned definitions and regardless of treatment or relationship to study product, must be recorded in the CRF by the investigator as soon as he/she is informed of the event.

The investigator must notify the sponsor of this event by faxing within 24 hours the "Serious Adverse Event (SAE) / Pregnancy / Likely Serious Adverse Effect or Requiring Medical Treatment Form" ("first notification", see appendix 17.2) with all the available information about the event to the sponsor's representative:

Name	:	Valérie ERMOSILLA, Pharmacien / PFDC Vigilances Manager
Fax	:	(33) 5 62 48 85 85
Phone	:	(33) 5 62 87 77 67
Adress	:	Pierre Fabre Dermo-Cosmétique<br>Hotel Dieu<br>2 rue Viguerie, BP 3071<br>31025 Toulouse Cedex 3<br>France

10.2.3. Follow-up of SAE

The investigator must use the "Serious Adverse Event (SAE) / Pregnancy / Likely Serious Adverse Effect or Requiring Medical Treatment Form" form ("follow-up") and collect the results of the carried out examinations and the reports of hospitalisation.

The serious adverse events must be followed up until resolution or stabilisation.

10.2.4. Suspicions of Serious adverse effects occurring after the study

Must be notified to the sponsor any serious adverse effect occurring at any time after the end of the study for the subject, which may be related to the study products in the investigator's opinion.

10.3. ADVERSE EVENTS RELATED TO THE STUDY PRODUCTS REQUIRING MEDICAL TREATMENT OR LIKELY TO BE SERIOUS

The adverse events related to the study products requiring medical treatment or likely to be serious must be notified by the investigator within 24 hours to the sponsor's representative (PFDC Vigilances Manager, see section 10.2.2) by faxing the "Serious Adverse Event (SAE) / Pregnancy / Likely Serious Adverse Effect or Requiring Medical Treatment Form".

These events must be followed until resolution.

Ex : allergic contact dermatitis

10.4. NEW EVENTS

Any new fact being sufficient in envisaging the modification of the use of the study products, in carrying out the research or relating to the research documents, or which may lead to, if necessary, a re-assessment of benefits and risks of the research, must be reported immediately by the investigator.

10.5. SPONSOR'S RESPONSIBILITIES FOR SAFETY REPORTING PURPOSES

Sponsor will submit to all investigators information that may affect the safety of persons undergoing research.

Sponsor will submit expedited and periodic reports to Ethics Committee taking into account local specific requirements.

11. DATA COLLECTION AND STUDY MONITORING

11.1. DATA COLLECTION

11.1.1. Case Report Form

Case report forms (CRFs) will be provided by the sponsor. They will contain all the data required by the protocol, except the computerised data (cf. § 11.1.3) which will be directly transferred to the sponsor's data base. Each CRF will be presented in triplicate. The original CRF page and the first copy will be stored by Pierre Fabre Dermo-Cosmetique at the end of the study. The last CRF copy will be stored by the investigator for at least 15 years after the end of the study or according to the local requirements.

Prior to the start of the study, the investigator will complete a "Delegation of significant study-related duties" form, showing the signatures and initials of any person who is authorised to make or change entries on the CRF.

Each CRF must be neatly filled in with a black-inked pen. All the information will be recorded from source documents onto the CRF by an authorised person, except for the overall efficacy assessed by the subject, by the investigator and the local tolerance of the product which will directly be reported onto the CRF.

All answer spaces on the forms must be completed, otherwise an explanation should be given on the corresponding form. If corrections are needed, a single line should be drawn through the wrong data, and the correct data should be written as close as possible to the crossed out data. These corrections should be initialled and dated by the authorised person who has corrected.

A CRF must be completed for each subject who will have received the study products.

The investigator is responsible for the management and accuracy of the information on the CRF. At each monitoring visit, the CRF(s) should be at the Clinical Research Associate's (CRA) disposition for review and collection.

The subject will be given four subject dairies during the study. In this diary, there is study products use conditions, treatment received or product applied on the hands and particular practice done concerning the skin. The subject has to note any omissions or changes in frequency of the products applications. He/she will also note if he/she observes any skin signs occurring between the visits. A first subject diary will be given to the subject by the investigator at the first visit (D1) that he will recover at the second visit (D90 ± 8 days). A second subject diary will be given to the parents by the investigator at the second visit that he will recover at the third visit (D180 ± 8 days). A third subject diary will be given to the parents by the investigator at the third visit that he will recover at the fourth visit (D270 ± 15 days). A fourth subject diary will be given to the parents by the investigator at the fourth visit that he will recover at the fifth visit (D360 ± 15 days).

At the second visit, the subject will also be given a questionnaire to assess his/her satisfaction as regard to the use of the test product. He/she will complete it during the second visit.

11.1.2. Source Documents

A source document is an original record or certified copy of an original record of clinical findings, observations or any other medical or paramedical activities performed during the clinical trial, necessary for the transcription and the verification of the collected data.

Source documents along with all other trial-related documents (copies of all "informed consent" forms, CRFs, product inventories and any correspondence related to the study) must be kept in the investigator's file throughout the study, and then, must be stored in the study centre's archives for a period of up to 15 years or as per local legal requirements. Hereafter, the research centre must contact the sponsor for instructions concerning the retention, return or destruction of the trial-related documents.

For further details see section 15.2.

11.1.3. Computerized data

Instrumental measurements (confocal microscopy, camera measurements, standardized photos) are directly recorded in electronic form and stored on computer media. Regular backups copy of data are performed throughout the study.

At the end of the study, all measures are merged and organized in a single file in a standard format to be determined.

The investigator provides two copies of this file to the sponsor: one of these copies acts as a witness file and is not subject to manipulation, the other copy is integrated into the database and therefore used as a data source for the different statistical analysis.

11.2. STUDY MONITORING

11.2.1. Monitoring visits

11.2.1.1. Site preselection visit

The aim of the preliminary visit will be to present the protocol to the investigator and his team, in a confidential manner, and to check the suitability of the human and material resources necessary for study implementation and the skills and experience of the investigator and his team.

This visit will also enable all the material, financial, administrative and regulatory aspects of the study to be addressed.

11.2.1.2. Initiation visit

The clinical research associate will check that all the materials and equipment necessary for the study implementation are available in the investigator's centre :

- Investigator's file
- Study protocol and appendices
- Case Report forms
- Informed consent forms
- Study products
- Study materials

11.2.1.3. Follow-up visits

The study follow-up visits will enable the clinical research associate to check compliance with the protocol and the conformity of the data collected with the source data.

The necessary corrections will be made by the investigator, then dated and initialled. The Clinical Research Associate will collect the original of the case report form, thus validated.

If required, the sponsor may request deferred corrections from the investigator, who will sign those corrections.

The visits will also enable checking of study products accountability.

11.2.1.4. Closing visit

The study termination visit will enable collection of the study products, case report forms and copies of the consent forms, if not done before. Study data storage (archiving) will also be checked.

11.2.2. Direct access to source documents

In agreeing to participate, the investigator undertakes to strictly comply with the study protocol, Good Clinical Practice and the national regulations. The investigator also guarantees the authenticity of the data collected in the context of the study and agrees to the legal provisions for study sponsor quality control.

In compliance with Good Clinical Practice, the data will undergo regular onsite control by the Clinical Research Associate.

The investigator undertakes to make him/herself available to the Clinical Research Associate and to make all the source documents available to the CRA for direct comparison with the CRF.

12. DATA MANAGEMENT

All clinical data related to the study will be collected and saved in a computerised database by the Data Management Department of PFB, except for the confocal data which will be collected and saved in a computerised database by the centre and under the supervision of the Data Manager of PFB according to the following procedures:

12.1. DATA ENTRY

Data entry screens will match the CRF.

Only after validation of the CRF by the CRA, a double data entry will be performed by the data entry operators of PFB using Oracle Clinical Software release 4.5.

Electronic data (confocal microscopy, camera measurements) not reported in the CRF by the investigator will be transferred by investigator into the database.

12.2. DATA REVIEW

After double data entry, a computerised data review will be performed by PFB. Consistency checks will be carried out using Oracle Clinical Software release 4.5 according to a consistency checks book.

Results of these checks will be sent to the study CRA and/or Monitor for resolution. Whenever required, queries will be submitted to the investigator for resolution and signature, and then tracked until corrections are entered and validated.

12.3. DATA STORAGE

The computer data files, as well as their modifications, will be saved and kept available upon request.
The data storage will be made in the PFB database for all data obtained from quotations and clinical data and in the centre database for confocal microscopy.
The confocal parameters will be stored in the centre; a copy of the pictures will be stored at PFDC.

12.4. DATABASE LOCK

The validated database will be locked upon request of the Data Manager of PFB following the completion of all steps required, i.e. : resolution of all queries, Clinical and Products Safety databases reconciliation and validation committee meeting.

13. STATISTICAL ANALYSIS

13.1. GENERAL CONSIDERATIONS

After the database lock and the randomisation code release, the statistical analysis will be performed par Pierre Fabre Biometrie using SAS® software.

13.2. SAMPLE SIZE

Because of the lack of data about laser associated with depigmenting treatment, the sample size has no statistical rationale.

The number of 30 subjects has been considered to be satisfactory for study analyse. Assuming that some subjects will be withdrawn from the study during the 12 months of follow up and others will be not entirely analysable, 40 subjects will be recruited for the study.

13.3. PROTOCOL DEVIATIONS

Major protocol deviations are defined as deviations liable to prevent or change the interpretation of the results of the study and therefore leading to the exclusion of the corresponding subjects from the Per Protocol (PP) population.

The following deviations will be considered as major :

- Non compliance with the inclusion or non-inclusion criteria
- Premature withdrawal or drop-out
- Intake of forbidden treatment
- No assessment of the primary efficacy criterion

- Non compliance with products application (if more than 30% of missed application between 2 consecutive visits)

All other protocol deviations will be considered as minor.

However, all deviations will require a blind review by the Validation Committee before data analysis.

The blind review process prepared by the clinical research associate, data-manager, study monitor and statistician will be completed by a meeting (Validation Committee) to be attended by at least the following :

- Coordinating Investigator,
- Study Monitor,
- Clinical Reasearch Associate,
- Data Manager,
- Statistician

The meeting will be documented by written and signed minutes that will act as the basis for data processing by Pierre Fabre Biométrie.

13.4. DATA SETS ANALYSED

The analyses will be conducted on the following data sets (as defined by the Validation Committee):

- The Safety set, composed of all randomised subjects having applied at least once the study products.
- The Full Analysis Set (FAS) composed of all randomised subjects having applied at least once the study products and with at least an evaluation of the primary criterion under products application
- The Per Protocol (PP) set, which is the subset of the Full Analysis data Set composed of all subjects without any major protocol deviations

13.5. HANDLING OF DROPOUTS AND MISSING DATA

The number and percentage of subjects who withdraw from the study after their randomisation will be provided for all treated subjects (safety set). All withdrawn subjects after randomisation will be further described regarding their time to dropout and reasons for withdrawal. Particular attention will be paid to the description of adverse events leading to premature withdrawal.

If the dropouts rate appears to affect significantly the statistical analysis, the main reasons for withdrawals should be cautiously examined, as well as their potential relationship to study products.

13.6. STATISTICAL METHODS

13.6.1. Description at baseline

At baseline, the population will be described as follow:

- demography: age, weight, height

- medical and surgical history

- concomitant diseases

- dermatological examination

Quantitative parameters will be described by groups using the followings: number of subjects, mean, standard deviation, minimum, median and maximum values.

Qualitative parameters will be described by group using frequencies and percentages.

13.6.2. Efficacy analysis

13.6.2.1. Primary criterion

The primary criterion is represented by the evolution of homogeneity of the skin colour assessed at T0 and T12 months by blinded evaluation from standardized photos, through 2 visual analogue scales (colour difference and affected surface, 0 to 10 for each), on both hands.

The sum of the two components (0 to 20) represents the homogeneity of the skin colour.

Primary analysis :

The change between T0 and T12 months will be compared between product using an analysis of covariance (ANCOVA) with product, site and sequence as fixed factor, subject as random factor and value at baseline as covariate.

This analysis will be made on the FAS.

Supportive analysis :

The same analysis will be made on PP set.

13.6.2.2. Secondary criteria

Criterion 1 is represented by the homogeneity of the skin colour assessed by clinical evaluation and by blinded evaluation (at T0, 3, 6, 9 and 12 months) from standardized photos through 2 visual analogue scales (colour difference and affected surface, 0 to 10 for each), on global hands, area treated by laser and area not treated by laser of both hands. The sum of the two components (0 to 20) represents the homogeneity of the skin colour.

Criterion 2 is represented by the lentigos number counted at T0, 3, 6, 9 and 12 months by blinded evaluation from standardized photos and by clinical evaluation, on global hands, area treated by laser and area not treated by laser of each hand.

Criterion 3 is represented by the Chromametry parameters (L*, a*, b*), using camera at T0, 3, 6, 9 and 12 months, on the target lesion treated by laser and the target lesion not treated by laser of each hand..

For criteria 1 to 3, changes between T0 with respectively T3, T6, T9 and T12 months will be compared between product using analysis of covariance which will use a likelihood-based Mixed-effects Model for Repeated Measures (MMRM) with product, site and sequence as fixed factor, subject as random factor and value at baseline as covariate. In case of significant difference for global model, appropriate contrasts will be used to determine differences between products at each time.

Criterion 4 is represented by Confocal parameters: pigment distribution, papillae contours, polycyclic papillary contours and enlarged interpapillary spaces defined by a 3 or 4 points scale, and epidermal thickness measured by confocal microscopy, at T0, 3, 6, 9 and 12 months on the target lesion treated by laser and the target lesion not treated by laser of each hand.

Changes between T0 with respectively T3, T6, T9 and T12 months will be compared between products. The Wilcoxon signed rank sum test will be used in order to compare products on the 4 parameters measured by scales. To compare thickness between both products, an analysis of covariance which will use a likelihood-based Mixed-effects Model for Repeated Measures (MMRM) with product, site and sequence as fixed factor, subject as random factor and value at baseline as covariate will be done. In case of significant difference for global model, appropriate contrasts will be used to determine differences between products at each time.

Criterion 5 is represented by the dynamic PGA at T3, 6, 9 and 12 months on the target lesion and peri-lesional target lesion treated by laser and the target lesion and peri-lesional target lesion not treated by laser of both hands. The dynamic PGA is defined through a 7 points scale (0 = completely cleared to 6 = appearance worse than baseline.

Criterion 6 is represented by Overall efficacy assessed by the Subject at 3 and 12 months on each global hand, on area treated by laser and area not treated by laser of each hand. The overall efficacy is defined through a 5 points scale (0 = completely improved to 4 = worse)

For criteria 5 and 6, comparisons between products will be made by Wilcoxon test using the Koch (16) method.

All statistics will be made on the whole hand for one part and on each area (treated and not treated by laser) for other part.

The satisfaction regarding to the use of D-pigment (conditions of use, organoleptic properties, effects...) will be assessed by the subject through a questionnaire after 3 months of daily applications. Results will be done by group through descriptive statistics with frequencies and percentages at the end of the study.

13.6.3. Safety Analysis

13.6.3.1. Adverse Events

The safety data set will be used to perform all analyses of the safety criteria.

Any adverse event having been reported during the study for a given subject will be classified as :

Any AE recorded during the treatment period will be regarded as Treatment Emergent (TE AE) if it was not already reported before the first intake of the randomised study product or if it began before and worsened during the randomised treatment period. Any AE starting in run-in/wash-out and going-on after the first intake of randomised study product will be qualified as emergent in case the relative information is missing or incomplete (ex: missing or incomplete end date in run-in/wash-out or no information regarding the intensity in run-in/wash-out and after the first intake of the randomised study product, separately). The frequency of subjects with at least one TE AE will be tabulated (n, %)

Non treatment emergent adverse events, i.e., any adverse event which occurs during the run-in / wash-out / run-out period and with no change in intensity during the randomised treatment period

Numbers and percentages of subjects with at least one reported treatment emergent adverse event will be tabulated by treatment group. Recurring adverse events for a given subject will only be counted once and only their most severe intensity will be tabulated.

13.6.3.2. Concomitant treatments

Concomitant treatments will be tabulated by treatment group.

13.7. COMPLIANCE

Estimation of compliance will be based on subject diary data and will be presented by application group on the Full Analysis Set. The analysis of compliance will be purely descriptive. No statistical test will be performed.

14. GENERAL ETHICAL CONSIDERATIONS

14.1. ETHICAL CONDITIONS

This study is performed in accordance with the principles stated in the Declaration of Helsinki and subsequent amendments and in accordance with the Good Clinical Practice Guideline (CPMP/ICH/135/95).

14.2. ETHICS COMMITTEE AND LEGAL REQUIREMENTS

All the documents required by National Regulations and any other informative documents that may be requested are submitted for review to an Ethics Committee (EC) whose procedures and operations meet the National Legal Requirements.

Depending on National Regulations, the application is submitted to the EC by the sponsor or by the investigator.

A copy of the formal written approval from the EC is provided to the sponsor (directly by the EC or via the investigator) with a list of names and qualifications of its members.

The request for authorisation by the Competent Authority or notification (depending on National Regulations) is carried out by the sponsor.

The screening of subjects does not start before the approval of the EC has been obtained and the study authorised by the Competent Authority (or notified to the Competent Authority, depending on the National Regulations).

14.3. SUBJECT'S INFORMATION LEAFLET AND INFORMED CONSENT FORM

An information must be given to the subjects before their decision to participate or abstain from participation.

This information is based on the elements set out in the Declaration of Helsinki and the ICH GCP Guideline. It must also describe the measures taken to safeguard subject's privacy and protection of personal data, according to European Directive 95/46 EC.

Restraints and risks must be explained, as well as the right to discontinue participation in the study at any stage, without affecting their further relationship with the investigator and/or their future care.

The written information and consent form must be submitted to the subject with an oral explanation. It must be agreed and signed by the subject before any study-related procedure starts.

This information and consent procedure is under the investigator's responsibility.

The information and consent document are made in triplicate*: the original copy is kept by the investigator, one copy is given to the subject and the last copy is kept by the Clinical Quality Assurance Unit of the sponsor in a sealed envelope at the end of study.

Specific areas of the sponsor's copy are not triplicated to avoid the reading of the subject's surname (except the first 3 letters), first name, address and signature.

If any information becomes available during the trial that may be relevant to the subject's willingness to keep on participating in the trial, an updated written informed consent must be submitted to the subject to confirm his agreement to continue participating.

14.4. PERSONAL DATA PROTECTION

All information from this study (excluding data from the informed consent) are entered into a computer under the sponsor's responsibility in accordance with the French law, "Loi Informatique et Libertés" (January 6, 1978 and subsequent amendments) and with the European Directive 95/46/EC.

14.5. INSURANCE POLICY

In accordance with the provisions of the law and the GCP, the sponsor Pierre Fabre Dermo-Cosmétique, has an insurance policy intended to guarantee against possible damage resulting from the research.

The studies and/or experiments performed on behalf of the sponsor Pierre Fabre Dermo-Cosmétique are specifically and expressly guaranteed.

It is advisable to underline that non compliance with the Research Legal Conditions is a cause for guarantee exclusion

15. ADMINISTRATIVE PROCEDURES

15.1. PROTOCOL AMENDMENT

Neither the investigator nor the sponsor may alter the protocol without the authorisation of the other party.

All changes to the protocol are subject to an amendment which must be dated and signed by both parties and must appear as an amendment to the protocol.

Substantial amendments are submitted for approval/authorization to EC and CA.

Urgent amendments are submitted for approval/authorisation to EC and CA but could be implemented immediately under specific conditions defined with the sponsor.

15.2. SOURCE DOCUMENTS, INVESTIGATOR’S FILE STORAGE

The investigator :

- keeps all trial-related documents in appropriate file folders. Records of subjects, original informed consent forms, source documents, case report forms, product inventory, EC and sponsor correspondence pertaining to the study must be kept on file.
- retains all documents relating to the screening (consent and investigation results) of all subjects included in the trial or not
- retains a list of the subjects names, addresses (and/or number of medical file), code numbers to allow checking of data reported on CRFs with those from source documents
- authorises direct access to source documents for monitoring, audits and inspections.

The trial-related documents must be retained as strictly confidential at the investigator's site for at least 15 years or according to local requirements, whatever is the longest after the completion or discontinuation of the trial.

15.3. END OF THE STUDY

15.3.1. Definition of the end of study

The end of study is the date of the last visit of the last subject undergoing the trial.

Any change to this definition during the study, for whatever reason, must be notified as a substantial amendment.

15.3.2. Early study termination

15.3.2.1. Early study termination decided by the sponsor

The sponsor may discontinue the study at any time for any of the following reasons :

- lack of recruitment
- deviations from Good Clinical Practice and/or regulations
- poor product safety, lack of efficacy
- new information that could jeopardise the subject’s safety
- stopping of development …

15.3.2.2. Early study termination decided by the Competent Authorities

The CA may suspend or prohibit a study if it considers that the conditions of authorisation are not being met or has doubt about the safety or scientific validity of the study.

15.4. AUDIT

The sponsor is responsible for making sure that both his representatives (monitor, CRA, ...) and the investigator fulfils the requirements as specified by the GCP Guideline.

An audit can be organised internally at the sponsor and at the investigational site where the CRFs are matched against source documents.

All study documentation must be directly accessible to auditors.

The practical conditions for the audit are discussed between the investigator and the Clinical Quality Assurance Department.

Oral information about the audit results are given to the investigator.

15.5. INSPECTION

The Health Authorities may audit any investigation site or the sponsor during the course of the study or after its completion, to verify the conduct of the study and quality of the data. The investigator must provide direct access to source documents.

15.6. CONFIDENTIALITY

The present materials (protocol, CRF) contain confidential information.

Except if agreed to in writing with the monitor, the investigators must hold such information confidential, and must not disclose it to others (except where required by applicable law).

15.7. CLINICAL STUDY REPORT

Data analysis, and clinical study report writing are under the sponsor’s responsibility.

Upon completion of the data analysis, a final report, including a review of the objectives and methods, a presentation and discussion of the results are drawn up and signed by the sponsor's representative(s) and the principal investigator.

15.8. STUDY RESULTS COMMUNICATION

Upon completion of the study, global results of the Research are communicated to the investigator. According to the Local Regulation, the subject can ask the investigator for the results

15.9. STUDY RESULTS PUBLICATION

The information and data collected during the conduct of this clinical study are considered confidential and are used by the sponsor in connection with the development of the study product. This information may be disclosed if deemed necessary by the sponsor.

To allow use of the information derived from this clinical study and to insure compliance with current regulations, the investigator must provide the sponsor with complete test results and all data collected during the study.

Only the sponsor may make study information available to physicians and to Regulatory Agencies, except as required by current regulations.

All the results of this study including data, reports, discoveries and inventions resulting from the study, are the property of the sponsor.

In the event that the sponsor chooses to publish study data, the manuscript must be provided to the author(s) at least 30 days prior to the expected date of submission to the intended publisher.

The investigator(s) can reserve the right to publish or present study data; if so, the manuscript or abstract must be provided to the sponsor for review at least 30 days prior to the expected date of submission to the intended publisher or of planned presentation.

In addition, if necessary, (the) investigator(s) shall withhold publication for an additional 60 days, to allow the filing of a patent application, or to allow the sponsor to take any measures he deems appropriate to establish and preserve his proprietary rights.

It is agreed that publication of study results by each site shall be made only as part of a publication of the study results obtained by all sites performing the protocol, once the study is completed and finalised.

The authors list is agreed by all investigators prior to publication. The names of the authors are provided according to their participation in the design of the protocol as well as their recruitment of eligible and analysable subjects.

16. REFERENCES

1. Cayce K A, McMichael A J, Feldman S R. Hyperpigmentation: an overview of the common afflictions. *Dermatol Nurs* 2004: **16**: 401–406.

2. Aoki H, Moro O, Tagami H, Kishimoto J. Gene expression profiling analysis of solar lentigo in relation to immunohistochemical characteristics. *Br J Dermatol* 2007: **156**: 1214–1223.

3. Gorlier C, Ortonne JP. Produits dépigmentants. *Encycl Méd Chir, Cosmétologie et Dermatologie esthétique*, 2001,50-210-A-10

4. Noblesse E, Nizard C, Cario-Andre M *et al.* Skin ultrastructure in senile lentigo. *Skin Pharmacol Physiol* 2006: **19**: 95–100.

5. Richtig E, Hofmann-Wellenhof R, Kopera D, El-Shabrawi-Caelen L, Ahlgrimm-Siess V. In vivo analysis of solar lentigines by reflectance confoncal microscopy before and after Q-switched ruby laser treatment. *Acta Derm Venerol* 2011; **91**:164-168

6. Fleischer AB Jr, Schwartzel EH, Colby SI, Altman DJ. The combination of 2% 4-hydroxyanisole (Mequinol) and 0.01% tretinoin is effective in improving the appearance of solar lentigines and related hyperpigmented lesions in two double-blind multicenter clinical studies. *J Am Acad Dermatol*. 2000 Mar; **42**(3):459-67.

7. Ortonne JP, Pandya AG, Lui H, Hexsel D. Treatment of solar lentigines. *J Am Acad Dermatol*. 2006 May; **54** (5 Suppl 2):S262-71.

8. Y.M. KIM et al J. Biol Chem., 25 (2002) 16340-16344

9. K. OHGUCHI et al Biochem Biophys. Res Comm., 307 (2003a) 861-863

10. G. VIELHABER et al IFSCC Magazine

11. B. Kasraee, G. Kaya, O. Sorg and J.H. Saurat

12. Haure MJ, Baudouin C, Ariès MF, *et al* J Invest Dermatol 122:A35, 2004

13. Khemis A, Cabou J, Dubois J, Ortonne JP. A randomized controlled study to evaluate the depigmenting activity of L-ascorbic acid plus phytic acid-serum vs. placebo on solar lentigines. *J Cosmet Dermatol*. 2011 Dec;**10**(4):266-72. doi: 10.1111/j.1473-2165.2011.00588.x.

14. Wang CC, Sue YM, Yang CH, Chen CK. A comparison of Q-switched alexandrite laser and intense pulsed light for the treatment of freckles and lentigines in Asian persons: a randomized, physician-blinded, split-face comparative trial. *J Am Acad Dermatol*. 2006 May;**54**(5):804-10.

15. Goldberg DJ. Complications in cutaneous laser surgery. Taylor & Francis; 1 edition (April 17, 2007)

16. Gary G. KOCH. The use of Non-Parametric Methods in the Statistical Analysis of the Two-Period Change-Over Design. *Biometrics.* 1972 Jun;**28**(2):577-84.

17. APPENDICES

17.1. DECLARATION OF HELSINKI

WORLD MEDICAL ASSOCIATION DECLARATION OF HELSINKI
Ethical Principles for Medical Research Involving Human Subjects

Adopted by the 18th WMA General Assembly, Helsinki, Finland, June 1964, and amended by the:
29th WMA General Assembly, Tokyo, Japan, October 1975
35th WMA General Assembly, Venice, Italy, October 1983
41st WMA General Assembly, Hong Kong, September 1989
48th WMA General Assembly, Somerset West, Republic of South Africa, October 1996
52nd WMA General Assembly, Edinburgh, Scotland, October 2000
53th WMA General Assembly, Washington 2002 (Note of Clarification on paragraph 29 added)
55th WMA General Assembly, Tokyo 2004 (Note of Clarification on Paragraph 30 added)
59th WMA General Assembly, Seoul, October 2008

A. INTRODUCTION

1. The World Medical Association (WMA) has developed the Declaration of Helsinki as a statement of ethical principles for medical research involving human subjects**,** including research on identifiable human material and data.

The Declaration is intended to be read as a whole and each of its constituent paragraphs should not be applied without consideration of all other relevant paragraphs.

2. Although the Declaration is addressed primarily to physicians, the WMA encourages other participants in medical research involving human subjects to adopt these principles.

3. It is the duty of the physician to promote and safeguard the health of patients, including those who are involved in medical research. The physician's knowledge and conscience are dedicated to the fulfilment of this duty.

4. The Declaration of Geneva of the WMA binds the physician with the words, “The health of my patient will be my first consideration,” and the International Code of Medical Ethics declares that, “A physician shall act in the patient's best interest when providing medical care.”

5. Medical progress is based on research that ultimately must include studies involving human subjects. Populations that are underrepresented in medical research should be provided appropriate access to participation in research.

6. In medical research involving human subjects, the well-being of the individual research subject must take precedence over all other interests.

7. The primary purpose of medical research involving human subjects is to understand the causes, development and effects of diseases and improve preventive, diagnostic and therapeutic interventions (methods, procedures and treatments). Even the best current interventions must be evaluated continually through research for their safety, effectiveness, efficiency, accessibility and quality.

8. In medical practice and in medical research, most interventions involve risks and burdens.

9. Medical research is subject to ethical standards that promote respect for all human subjects and protect their health and rights. Some research populations are particularly vulnerable and need special protection. These include those who cannot give or refuse consent for themselves and those who may be vulnerable to coercion or undue influence.

10. Physicians should consider the ethical, legal and regulatory norms and standards for research involving human subjects in their own countries as well as applicable international norms and standards. No national or international

ethical, legal or regulatory requirement should reduce or eliminate any of the protections for research subjects set forth in this Declaration.

B. PRINCIPLES FOR ALL MEDICAL RESEARCH

11. It is the duty of physicians who participate in medical research to protect the life, health, dignity, integrity, right to self-determination, privacy, and confidentiality of personal information of research subjects.

12. Medical research involving human subjects must conform to generally accepted scientific principles, be based on a thorough knowledge of the scientific literature, other relevant sources of information, and adequate laboratory and, as appropriate, animal experimentation. The welfare of animals used for research must be respected.

13. Appropriate caution must be exercised in the conduct of medical research that may harm the environment.

14. The design and performance of each research study involving human subjects must be clearly described in a research protocol. The protocol should contain a statement of the ethical considerations involved and should indicate how the principles in this Declaration have been addressed. The protocol should include information regarding funding, sponsors, institutional affiliations, other potential conflicts of interest, incentives for subjects and provisions for treating and/or compensating subjects who are harmed as a consequence of participation in the research study. The protocol should describe arrangements for post-study access by study subjects to interventions identified as beneficial in the study or access to other appropriate care or benefits.

15. The research protocol must be submitted for consideration, comment, guidance and approval to a research ethics committee before the study begins. This committee must be independent of the researcher, the sponsor and any other undue influence. It must take into consideration the laws and regulations of the country or countries in which the research is to be performed as well as applicable international norms and standards but these must not be allowed to reduce or eliminate any of the protections for research subjects set forth in this Declaration. The committee must have the right to monitor ongoing studies. The researcher must provide monitoring information to the committee, especially information about any serious adverse events. No change to the protocol may be made without consideration and approval by the committee.

16. Medical research involving human subjects must be conducted only by individuals with the appropriate scientific training and qualifications. Research on patients or healthy volunteers requires the supervision of a competent and appropriately qualified physician or other health care professional. The responsibility for the protection of research subjects must always rest with the physician or other health care professional and never the research subjects, even though they have given consent.

17. Medical research involving a disadvantaged or vulnerable population or community is only justified if the research is responsive to the health needs and priorities of this population or community and if there is a reasonable likelihood that this population or community stands to benefit from the results of the research.

18. Every medical research study involving human subjects must be preceded by careful assessment of predictable risks and burdens to the individuals and communities involved in the research in comparison with foreseeable benefits to them and to other individuals or communities affected by the condition under investigation.

19. Every clinical trial must be registered in a publicly accessible database before recruitment of the first subject.

20. Physicians may not participate in a research study involving human subjects unless they are confident that the risks involved have been adequately assessed and can be satisfactorily managed. Physicians must immediately stop a study when the risks are found to outweigh the potential benefits or when there is conclusive proof of positive and beneficial results.

21. Medical research involving human subjects may only be conducted if the importance of the objective outweighs the inherent risks and burdens to the research subjects.

22. Participation by competent individuals as subjects in medical research must be voluntary. Although it may be appropriate to consult family members or community leaders, no competent individual may be enrolled in a research study unless he or she freely agrees.

23. Every precaution must be taken to protect the privacy of research subjects and the confidentiality of their personal information and to minimize the impact of the study on their physical, mental and social integrity.

24. In medical research involving competent human subjects, each potential subject must be adequately informed of the aims, methods, sources of funding, any possible conflicts of interest, institutional affiliations of the researcher, the anticipated benefits and potential risks of the study and the discomfort it may entail, and any other relevant aspects of the study. The potential subject must be informed of the right to refuse to participate in the study or to withdraw consent to participate at any time without reprisal. Special attention should be given to the specific information needs of individual potential subjects as well as to the methods used to deliver the information. After ensuring that the potential subject has understood the information, the physician or another appropriately qualified individual must then seek the potential subject's freely-given informed consent, preferably in writing. If the consent cannot be expressed in writing, the non-written consent must be formally documented and witnessed.

25. For medical research using identifiable human material or data, physicians must normally seek consent for the collection, analysis, storage and/or reuse. There may be situations where consent would be impossible or impractical to obtain for such research or would pose a threat to the validity of the research. In such situations the research may be done only after consideration and approval of a research ethics committee.

26. When seeking informed consent for participation in a research study the physician should be particularly cautious if the potential subject is in a dependent relationship with the physician or may consent under duress. In such situations the informed consent should be sought by an appropriately qualified individual who is completely independent of this relationship.

27. For a potential research subject who is incompetent, the physician must seek informed consent from the legally authorized representative. These individuals must not be included in a research study that has no likelihood of benefit for them unless it is intended to promote the health of the population represented by the potential subject, the research cannot instead be performed with competent persons, and the research entails only minimal risk and minimal burden.

28. When a potential research subject who is deemed incompetent is able to give assent to decisions about participation in research, the physician must seek that assent in addition to the consent of the legally authorized representative. The potential subject's dissent should be respected.

29. Research involving subjects who are physically or mentally incapable of giving consent, for example, unconscious patients, may be done only if the physical or mental condition that prevents giving informed consent is a necessary characteristic of the research population. In such circumstances the physician should seek informed consent from the legally authorized representative. If no such representative is available and if the research cannot be delayed, the study may proceed without informed consent provided that the specific reasons for involving subjects with a condition that renders them unable to give informed consent have been stated in the research protocol and the study has been approved by a research ethics committee. Consent to remain in the research should be obtained as soon as possible from the subject or a legally authorized representative.

30. Authors, editors and publishers all have ethical obligations with regard to the publication of the results of research. Authors have a duty to make publicly available the results of their research on human subjects and are accountable for the completeness and accuracy of their reports. They should adhere to accepted guidelines for ethical reporting. Negative and inconclusive as well as positive results should be published or otherwise made publicly available. Sources of funding, institutional affiliations and conflicts of interest should be declared in the publication. Reports of research not in accordance with the principles of this Declaration should not be accepted for publication.

C. ADDITIONAL PRINCIPLES FOR MEDICAL RESEARCH COMBINED WITH MEDICAL CARE

31. The physician may combine medical research with medical care only to the extent that the research is justified by its potential preventive, diagnostic or therapeutic value and if the physician has good reason to believe that participation in the research study will not adversely affect the health of the patients who serve as research subjects.

32. The benefits, risks, burdens and effectiveness of a new intervention must be tested against those of the best current proven intervention, except in the following circumstances:

- The use of placebo, or no treatment, is acceptable in studies where no current proven intervention exists; or
- Where for compelling and scientifically sound methodological reasons the use of placebo is necessary to determine the efficacy or safety of an intervention and the patients who receive placebo or no treatment will not be subject to any risk of serious or irreversible harm. Extreme care must be taken to avoid abuse of this option.

33. At the conclusion of the study, patients entered into the study are entitled to be informed about the outcome of the study and to share any benefits that result from it, for example, access to interventions identified as beneficial in the study or to other appropriate care or benefits.

34. The physician must fully inform the patient which aspects of the care are related to the research. The refusal of a patient to participate in a study or the patient’s decision to withdraw from the study must never interfere with the patient-physician relationship.

35. In the treatment of a patient, where proven interventions do not exist or have been ineffective, the physician, after seeking expert advice, with informed consent from the patient or a legally authorized representative, may use an unproven intervention if in the physician's judgement it offers hope of saving life, re-establishing health or alleviating suffering. Where possible, this intervention should be made the object of research, designed to evaluate its safety and efficacy. In all cases, new information should be recorded and, where appropriate, made publicly available.

17.2. SERIOUS ADVERSE EVENT (SAE) / PREGNANCY / LIKELY SERIOUS ADVERSE EFFECT OR REQUIRING MEDICAL TREATMENT FORM

PIERRE FABRE DERMO-COSMETIQUE	PROTOCOL N°RV4280A2012607	SUBJECT'S CODE<br>|_0_|_1_| |___|___| Centre number Subject's number	1/3

Interventional study ❑ yes ❑ no

SERIOUS ADVERSE EVENT (SAE) / PREGNANCY / LIKELY SERIOUS ADVERSE EFFECT OR REQUIRING MEDICAL TREATMENT FORM - COSMETIC STUDY

To be faxed within 24H to Cosmetovigilance Manager PFDC :

Mrs Valérie ERMOSILLA
Phone n°: +33(0)5.62.87.77.67
Fax n°: +33(0)5.62.48.85.85

Transmission date |__|__||__|__||__|__| (d d m m y y) Investigator name ____________ Country ____________

Event n° |__|__| First notification ❑ Follow up ❑

Subject characteristics

Name : |__|__|__|	First name : |__|__|	Birth date : |__|__| |__|__| |__|__| (d d m m y y)	Sexe : |__|	1 = M<br>2 = F

Type of event

❑ Serious Adverse Event (SAE) *	*In case of SAE, please note if the event resulted in :
❑ Pregnancy	❑ Death (whatever may be the cause)
❑ Likely serious adverse effect	❑ Congenital anomaly or birth defect
❑ Adverse effect requiring medical treatment	❑ Life-threatening
	❑ Hospitalisation or extension thereof
Beginning date : |__|__| |__|__| |__|__| (d d m m y y)	❑ Persistent or significant disability/incapacity
Ending date : |__|__| |__|__| |__|__| (d d m m y y)	

Description of the event

Description (clinical cause, previous relevant history, if available complementary tests results): ____
____
____

Diagnosis in relation to signs and symptoms : ____

Allergic reaction : ❑ yes ❑ no
If yes,
- objective and concomitant subjective signs: ____

- Localisation of the reaction :
  - ✓ application area ❑ to specify : ____________________________
  - ✓ remote ❑ to specify : ____________________________

- Does the effect justify a patch testing : ❑ yes ❑ no
- If yes, does the subject accept to undertake patch-tests : ❑ yes ❑ no

Formulaire - COS_002550 - Version : 3 - Date d'application : 23/11/2010

Document réalisé à partir du modèle bureautique « Document » référencé COS004661.1
1/3

PIERRE FABRE DERMO-COSMETIQUE	PROTOCOL N°RV4280A2012607	SUBJECT'S CODE<br>|_0_|_1_| |__|__| Centre number Subject's number	2/3

Details about the study product and/or the associated product

**Study product**	**Associated product***
Product(s) code or name: ___________________	Product(s) code or name: ___________________
Time of occurrence of the event:	Time of occurrence of the event:
❑ During the selection period	❑ During the selection period
❑ During the administration of the product(s)	❑ During the administration of the product(s)
❑ After the administration of the product(s)	❑ After the administration of the product(s)
Date of first use : |__|__| |__|__| |__|__| *d d m m y y*	Date of first use : |__|__| |__|__| |__|__| *d d m m y y*
Date of last use <u>before</u> the occurrence of the event :<br>|__|__| |__|__| |__|__| *d d m m y y*	Date of last use <u>before</u> the occurrence of the event :<br>|__|__| |__|__| |__|__| *d d m m y y*
Frequency of use: ___________________	Frequency of use: ___________________
Quantity applied: ___________________	Quantity applied: ___________________
Application area: ___________________	*Application area:* ___________________

** To fill in only if the event might be related to the associated product*

Concomitant treatments (do not include treatment(s) given for the event)

Name	Daily dose	Start date *(ddmmyy)*	On going	Stop date *(ddmmyy)*	Route of admin.	indication
		___/___/___	❑	___/___/___		
		___/___/___	❑	___/___/___		
		___/___/___	❑	___/___/___		

Action taken following the event

<u>**-Related to the study product**</u>

❑ Change in the protocol or use, specify : ___________________

Date of change : |__|__| |__|__| |__|__|
d d m m y y

Duration : ___________________

❑ Temporary interruption of use, specify duration : ___________________

Date of reconduction : |__|__| |__|__| |__|__|
d d m m y y

❑ Stop of use Ending date : |__|__| |__|__| |__|__|
d d m m y y

❑ No change

<u>**- The event has resulted in**</u>

❑ Complementary tests:___________________
❑ Prescription of treatments, to specify (names, dosages, duration): ___________________
❑ Discontinuation of concomitant treatments, to specify (names): ___________________
❑ Urgent medical operations, to specify: ___________________
❑ Cessation from work, to specify: ___________________
❑ Premature withdrawal of the study. If yes, date: |__|__| |__|__| |__|__|
d d m m y y

❑ Other, to specify: ___________________

Formulaire - COS_002550 - Version : 3 - Date d'application : 23/11/2010

PIERRE FABRE DERMO-COSMETIQUE	PROTOCOL N°RV4280A2012607	SUBJECT'S CODE<br>|_0_|_1_| |__|__| Centre number Subject's number	3/3

Blind code broken

Blind code broken if double blind study : ❑ yes ❑ no
If yes, results : ____

Outcome

❑ Not recovered/Not resolved ❑ Recovering/Resolving ❑ Recovered/Resolved
❑ Recovered/Resolved with sequelae ❑ Death ❑ Unknown

Investigator's opinion on relationship of event with the study products/associated products

Relation with a study product ❑ Not suspected ❑ Suspected ❑ Insufficient data

If suspected and in case of an open-blinded comparative study, please specify which one of the products is suspected:
❑ Tested product ❑ Comparative product

Relation with an associated product❑ Not suspected ❑ Suspected ❑ Insufficient data

Other: ____

Attach copies of all investigations and hospital discharge forms, etc…

Investigator name : Signature :
